# Supplementary material for: A Novel Expression Domain of extradenticle Underlies the Evolutionary Developmental Origin of the Chelicerate Patella
Source: Mol Biol Evol. 2024 Sep 5;41(9):msae188. doi: 10.1093/molbev/msae188 (PMC11422720; doi:10.1093/molbev/msae188)
Supplement: msae188_Supplementary_Data [file msae188_supplementary_data.zip › Supplementary.File_S5.pdf]

Electronic Supplementary Material

Benjamin C. Klementz[1,2*], Georg Brenneis[3], Isaac A. Hinne[4], Ethan M. Laumer[1,2], Sophie M. Neu[1,2], Grace M. Hareid[1,2], Guilherme Gainett[1,5], Emily V.W. Setton[1,6], Catalina Simian[7], David E. Vrech[7], Isabella Joyce[8], Austen A. Barnett[8], Nipam H. Patel[9], Mark S. Harvey[10], Alfredo V. Peretti[7], Monika Gulia-Nuss[4], Prashant P. Sharma[1,2]

[1] Department of Integrative Biology, University of Wisconsin-Madison, Madison, Wisconsin, USA.
[2] University of Wisconsin-Madison Zoological Museum, Madison, Wisconsin, USA.
[3] Unit Integrative Zoologie, Department Evolutionsbiologie, Universität Wien, Vienna, Austria.
[4] Department of Biochemistry and Molecular Biology, University of Nevada, Reno, Nevada, USA.
[5] Department of Systems Biology, Harvard Medical School, Boston, Massachusetts, USA.
[6] Department of Ecology and Evolutionary Biology, Cornell University, Ithaca, New York, USA.
[7] Laboratorio de Biología Reproductiva y Evolución, Consejo Nacional de Investigaciones Científicas Técnicas (CONICET), Instituto de Diversidad y Ecología Animal (IDEA), Córdoba, Argentina.
[8] Department of Biology, DeSales University, Center Valley, Pennsylvania, USA.
[9] Marine Biological Laboratory, Woods Hole, Massachusetts, USA.
[10] Western Australian Museum, Welshpool, Western Australia, Australia.

*Correspondence: bklementz@wisc.edu

This PDF file includes:

Supplementary Figures S1 to S8
Supplementary Tables S1-S20
Supplementary Methods
Supplementary References

Supplementary Figures

Figure S1. Wild type expression of proximodistal patterning genes in representative stages of appendage formation in *P. opilio.* All embryos in ventral view with anterior toward the right. Each row represents a single embryo with single-channel visualization of Hoechst nuclear counterstaining (cyan), and expression of *Po-dac* (yellow), *Po-exd* (magenta), and *Po-Dll* (red).

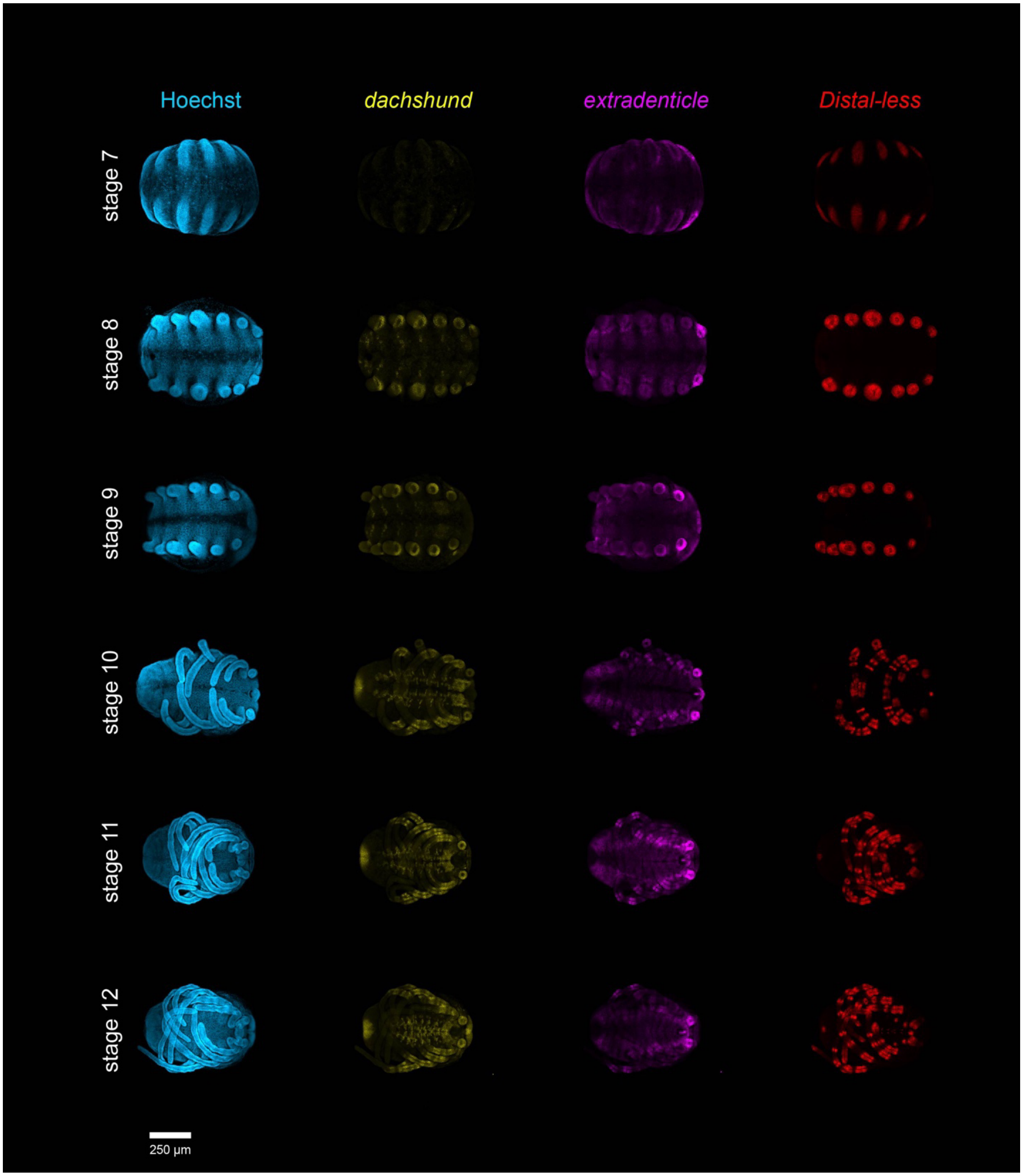

Figure S2. Wild type expression of proximodistal patterning genes in representative stages of appendage formation in *P. opilio.* All embryos in lateral view with anterior toward the right. Each row represents a single embryo with single-channel visualization of Hoechst nuclear counterstaining (cyan), and expression of *Po-dac* (yellow), *Po-exd* (magenta), and *Po-Dll* (red).

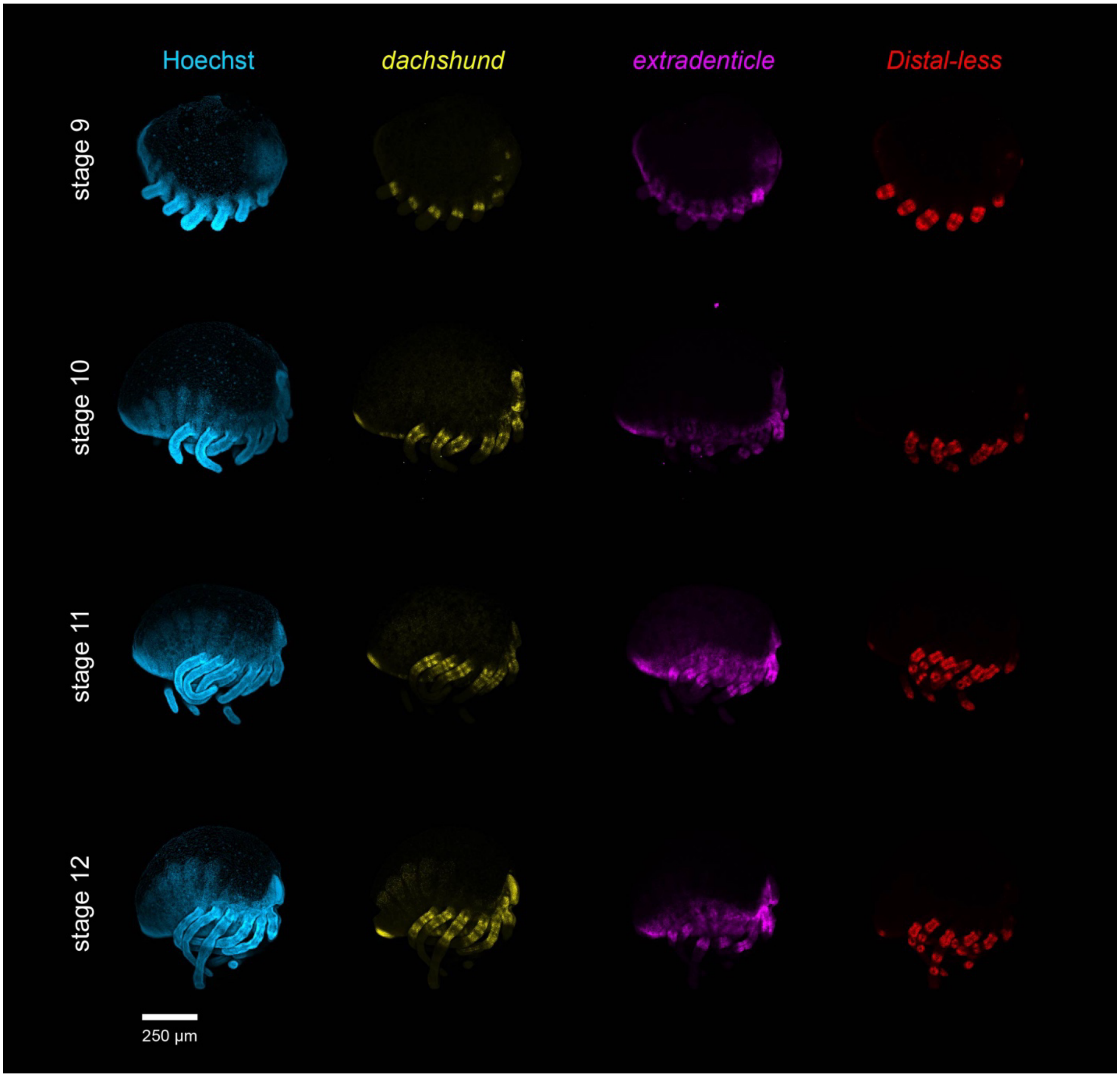

Figure S3. Wild type expression of proximodistal patterning genes in dissected appendages during representative stages of appendage formation in *P. opilio.* Appendages represent those in Figure 3 with demultiplexed single-channel visualization of Hoechst nuclear counterstaining (cyan), and expression of *Po-dac* (yellow), *Po-exd* (magenta), and *Po-Dll* (red). All appendages are in lateral view with distal to the right.

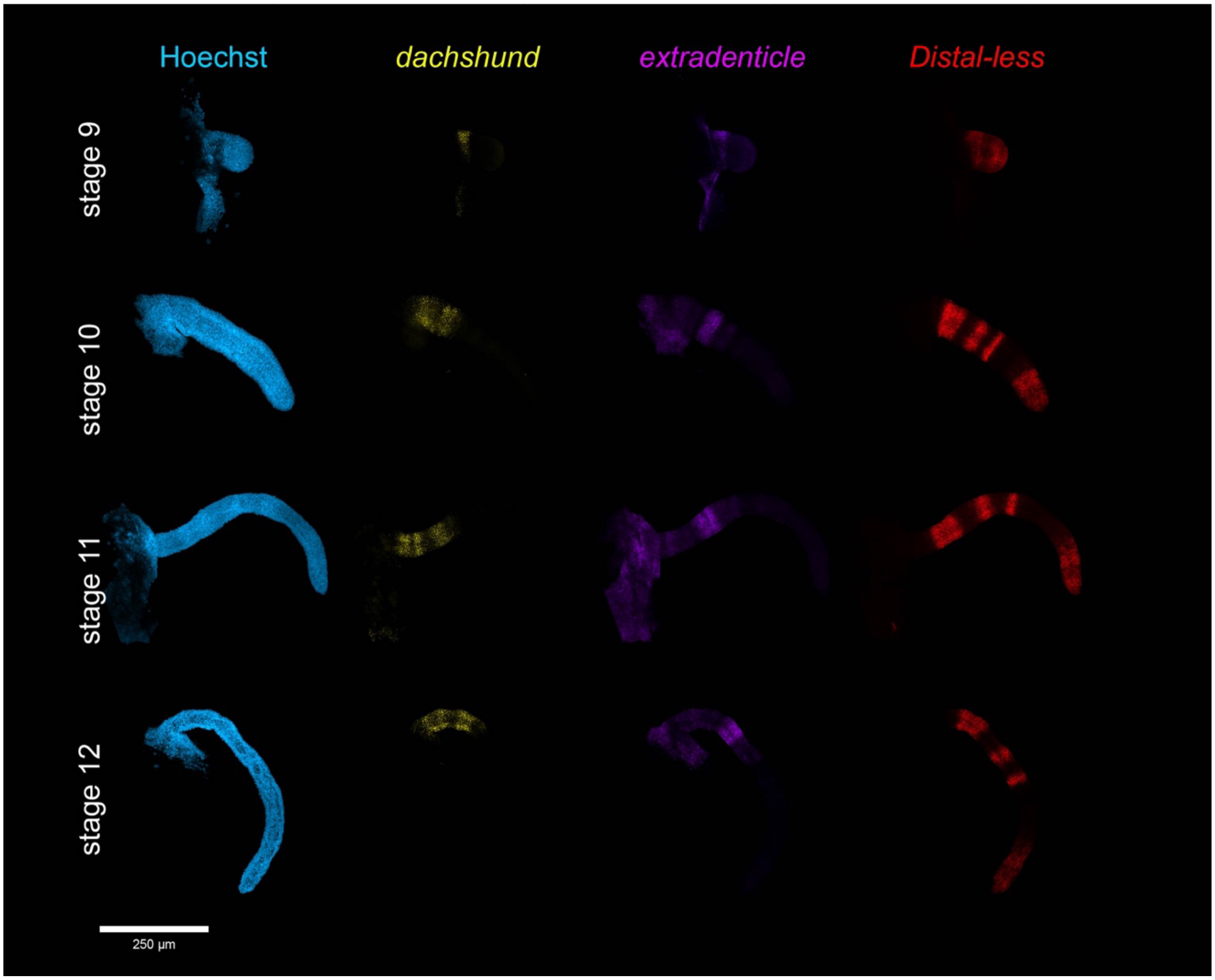

Figure S4. Validation of on-target *Po-exd* RNAi-mediated knockdown. Each row corresponds to an individual negative control (**a-a''**) or *Po-exd* RNAi embryo (**b-b''**). Embryos presented in ventral view with anterior toward the top. (**a, b**) Hoechst nuclear counterstaining (cyan). (**a', b'**) Expression of *Po-exd* (magenta). (**a'', b''**) Expression of *Po-Dll* (red).

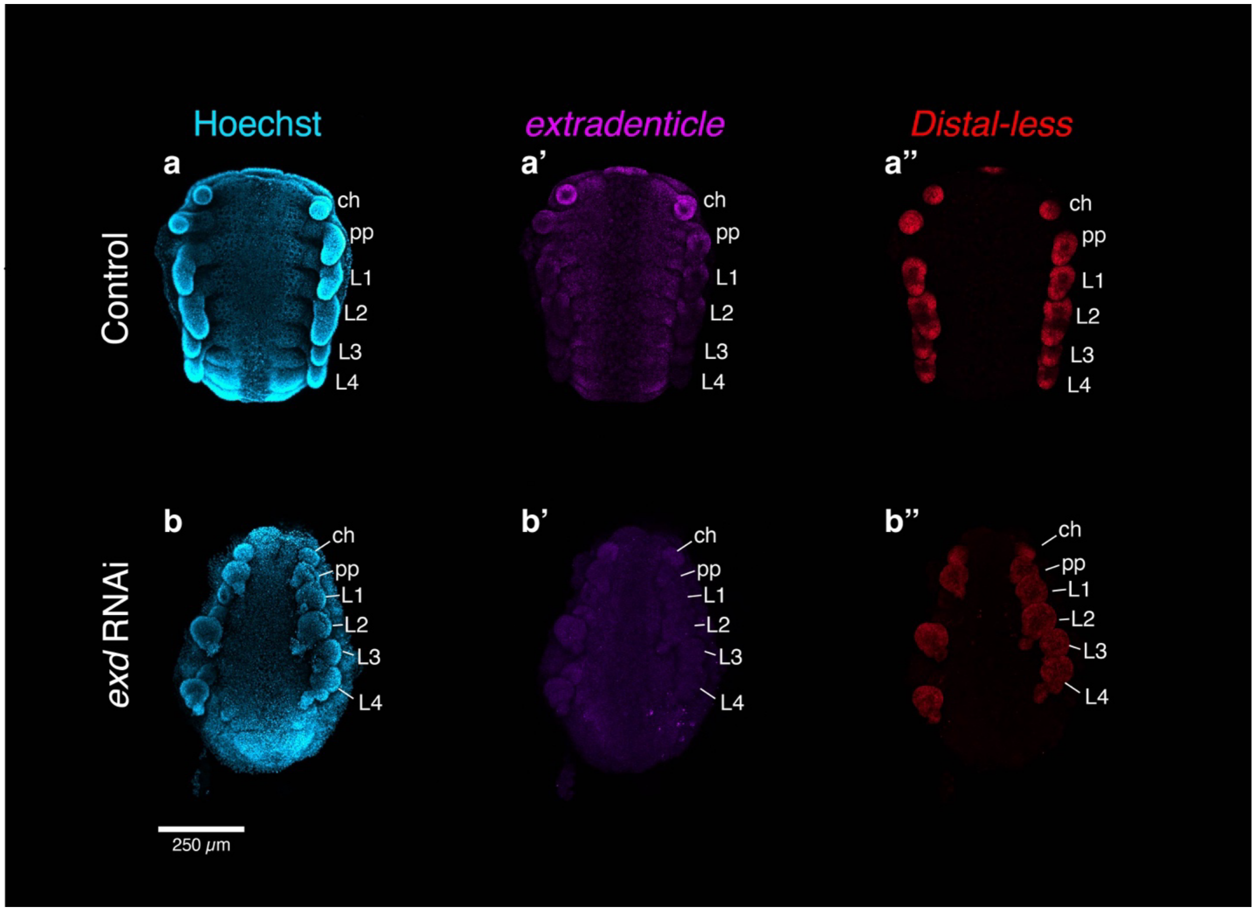

Figure S5. Knockdown of *Po-N* yields additional defects in central nervous system development, visualized via expression of *Po-dac.* Each row corresponds to an individual negative control (**a-a'''**) or *Po-N* RNAi embryo (**b-b'''**). Embryos presented in ventral view with anterior toward the top. (**A, B**) Hoechst nuclear counterstaining (cyan). (**a', b'**) Expression of *Po-dac* (yellow). (**a'', b''**) Expression of *Po-exd* (magenta). (**a''', b'''**) Expression of *Po-N* (orange). Abbreviations: lb, labrum; ch, chelicera; pp, pedipalp; L1, leg one; L2, leg two; L3, leg three; L4, leg four.

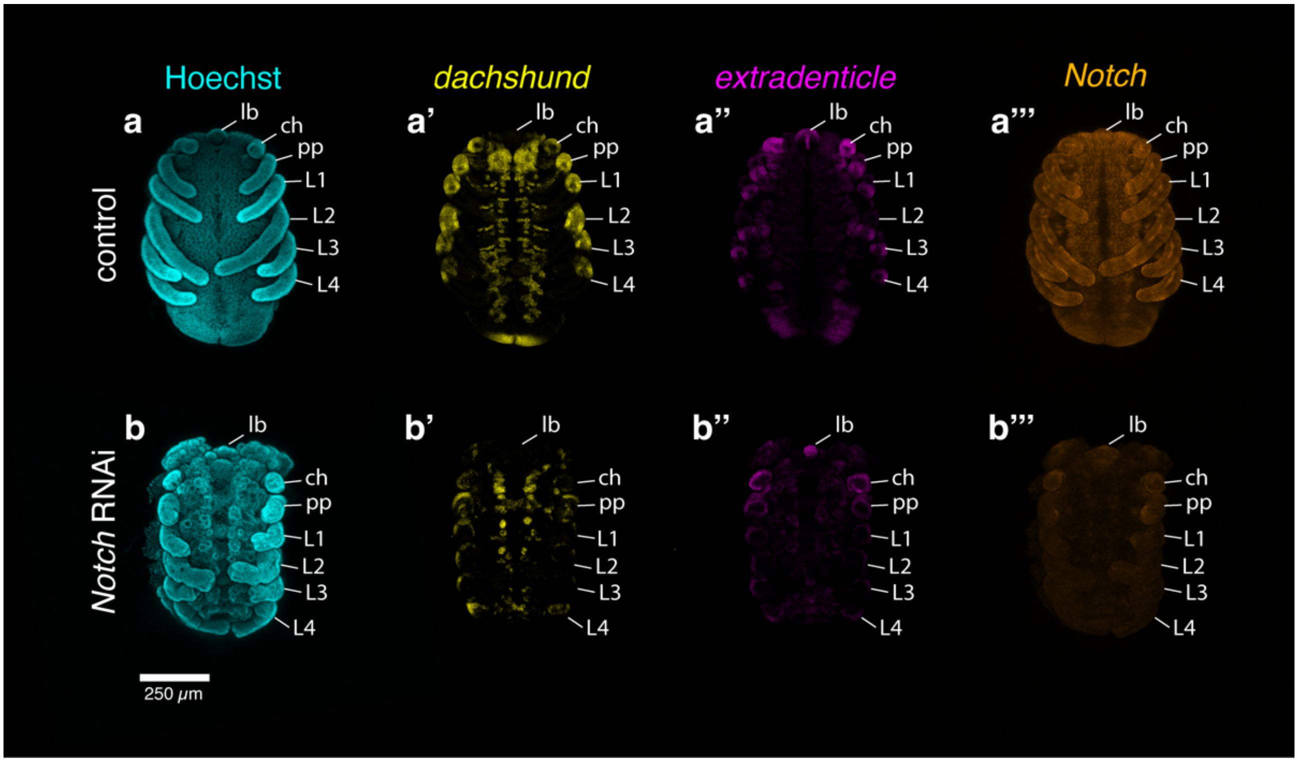

Figure S6. Range of observed segmental defects, visualized via expression of *Po-en* (green), following *Po-N* RNAi treatments. (**a**) Negative control embryo with wild type morphology and expression of *Po-en* in iterative stripes in the posterior compartment of all body segments and appendages. (**b**) Weak phenotype following *Po-N* RNAi. Embryo retains truncated, abnormal segmental stripes of *Po-en* in the body, but restricted expression in the appendages to distal termini. (**c**) Strong phenotype following *Po-N* RNAi. *Po-en* expression is detected only in the distal termini of appendages.

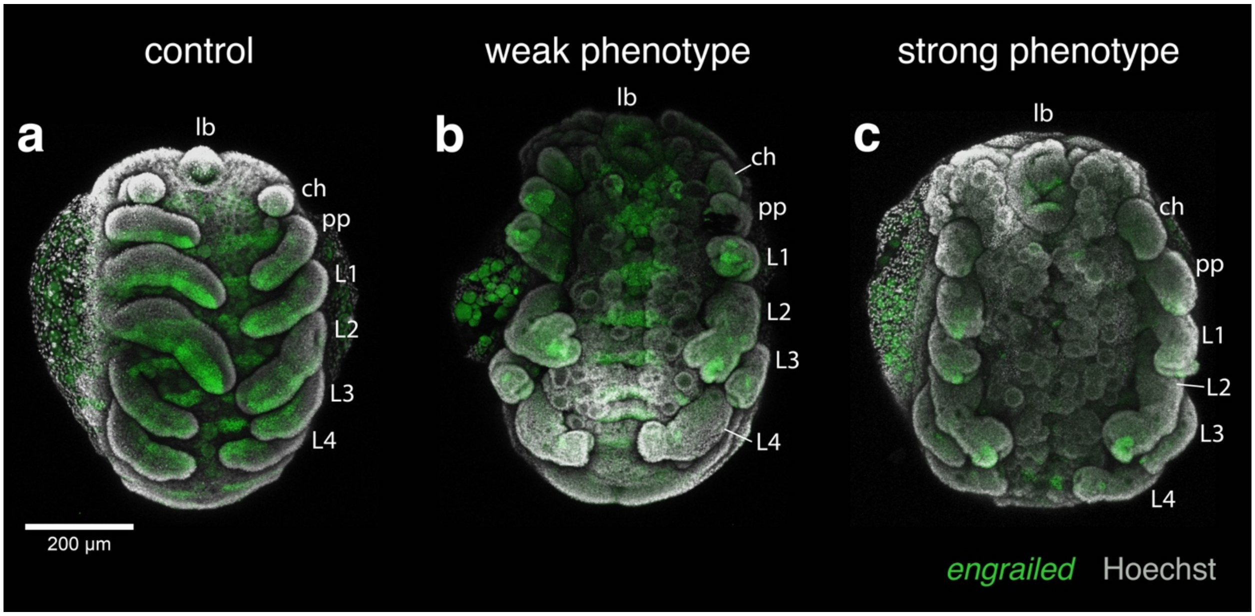

Figure S7. Dissected appendages of *Po-N* RNAi embryos demonstrate prominent reductions in *Po-exd* expression, but not *Po-dac*. Rows correspond to individual appendages from negative control (**a-a'''**) or *Po-N* RNAi embryos (**b-b''', c-c''**). All appendages in lateral view with distal to the right. (**a-c**) Hoechst nuclear counterstaining (gray) with merged expression of *Po-dac* (yellow) and *Po-exd* (magenta). (**a'-c'**) Single-channel visualization of Hoechst counterstaining. (**a''-b''**) Single-channel expression of *Po-dac*. (**a'''-c''**) Single-channel expression of *Po-exd*. Embryo in (**c-c''**) was not surveyed for expression of *Po-dac*.

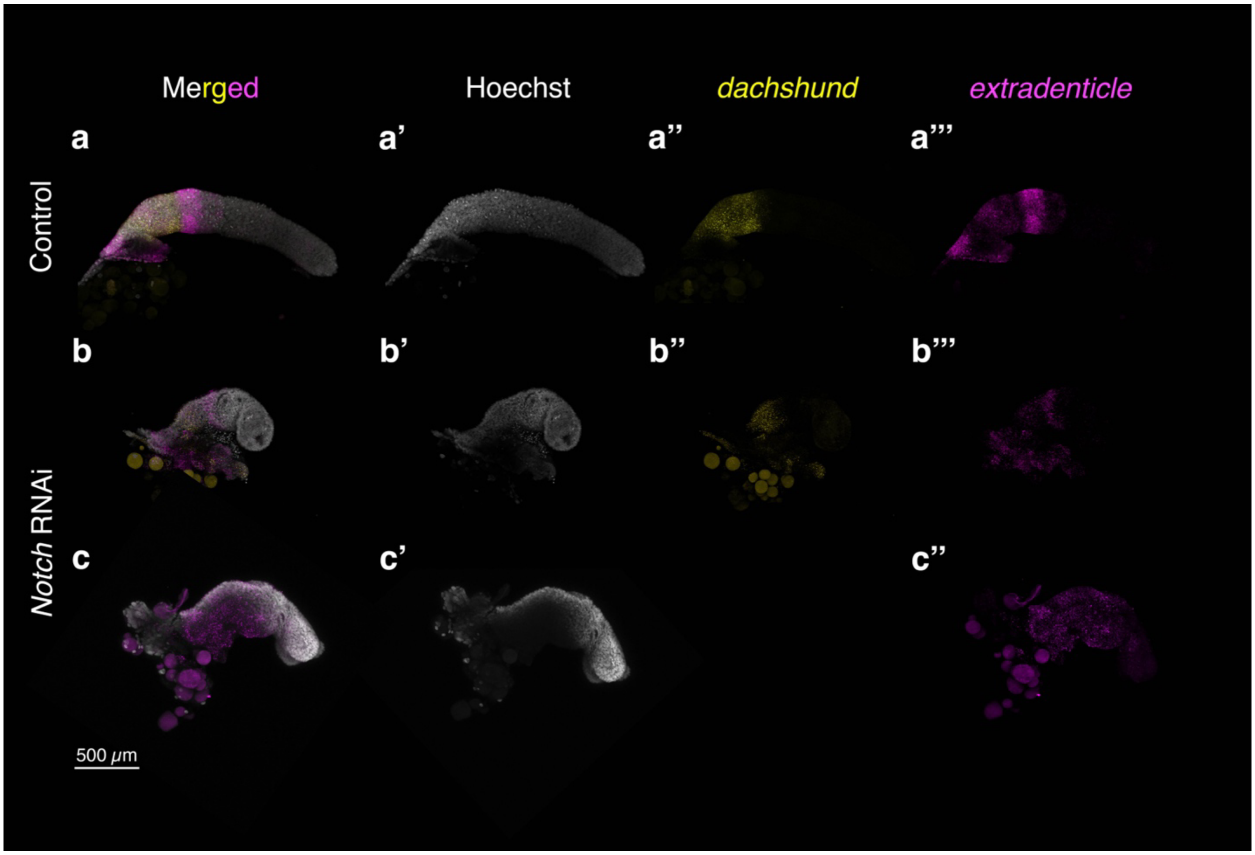

Figure S8. Overview of *Pl-exd, Pl-dac,* and *Pl-Dll* expression during postembryonic development of *P. litorale.* All images show ventral views. One leg of instar IV (**d, e**) has been virtually removed (dashed line). Arrows: distal boundary of *Pl-dac* domain. Arrowheads: *Pl-exd* expression distal to the *Pl-dac* domain. Asterisks indicate regions of damaged tissue. (**a**) Late instar II. (**b**) Early instar III. (**c**) Late instar III. (**d**) Early instar IV. (**e**) Late instar IV. Abbreviations: ch, chelicera; L, leg; ov, ovigeral larval limb; pp, pedipalpal larval limb.

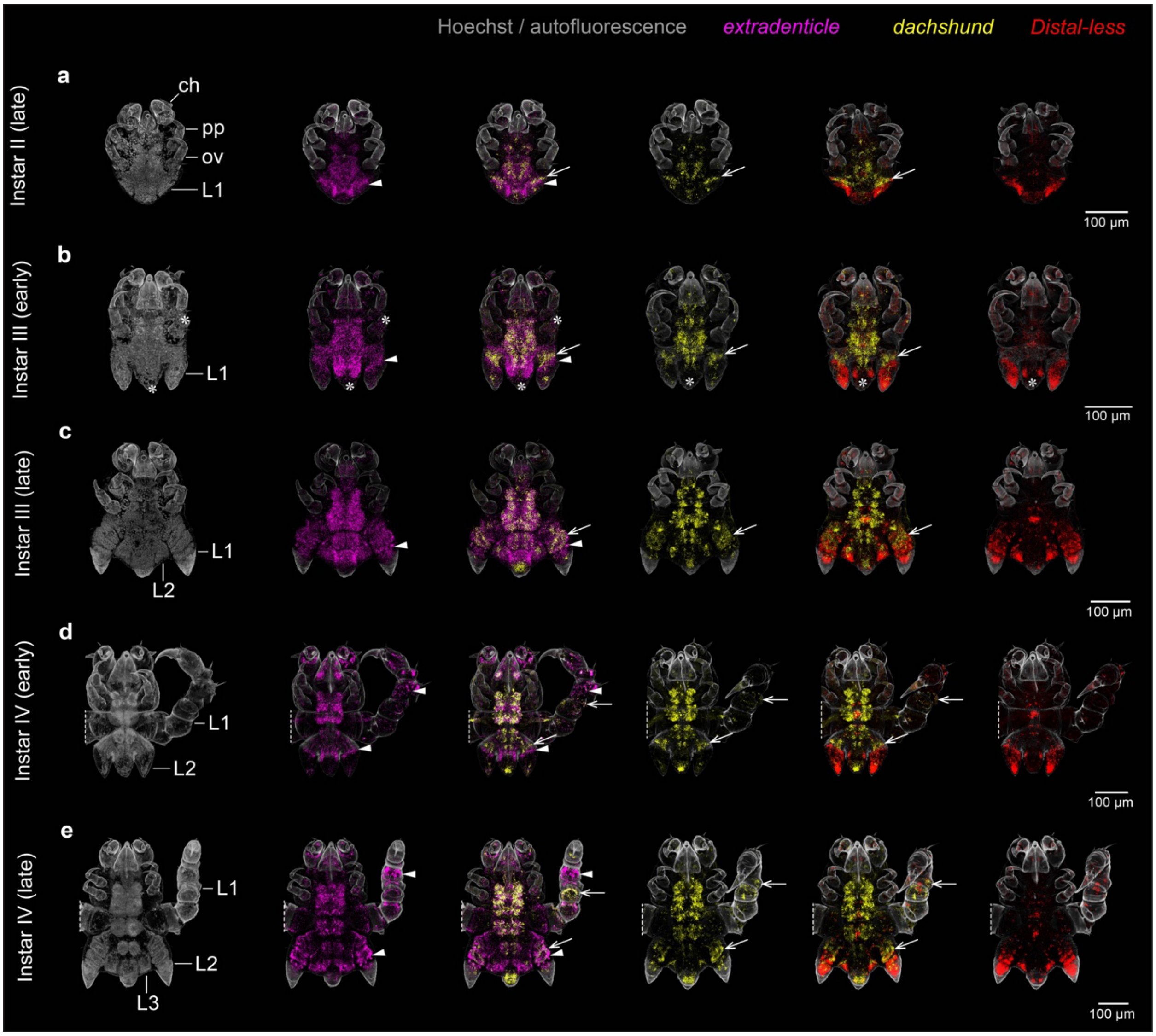

Figure S9. Combinatorial expression of *Is-dac* paralogs allows for diagnosis of podomere identity. (**a**) Stage 10 *I. scapularis* embryo with multiplexed Hoechst nuclear counterstaining (gray), expression of *Is-exd* (magenta), and both *Is-dacA* and *Is-dacB* (yellow). Note the absence of *Is-exd* expression distal to *Is-dac* paralogs. (**a'**) Same embryos as in (**a**) with isolated expression of *Is-exd*, *Is-dacA,* and *Is-dacB.* (**b**) Stage 24 *I. scapularis* embryo with Hoechst nuclear counterstaining (cyan) and combinatorial *Is-dacA* and *Is-dacB* expression (yellow). Note prominent podomere boundaries and refinement of *Is-dac* expression to the distal compartments of the trochanter, basifemur, and telofemur. Abbreviations: cx, coxa; tr, trochanter; bfe, basifemur; tfe, telofemur; ti, tibia; bta, basitarsus; tta, telotarsus; L1, leg one; L2, leg two; L3, leg three.

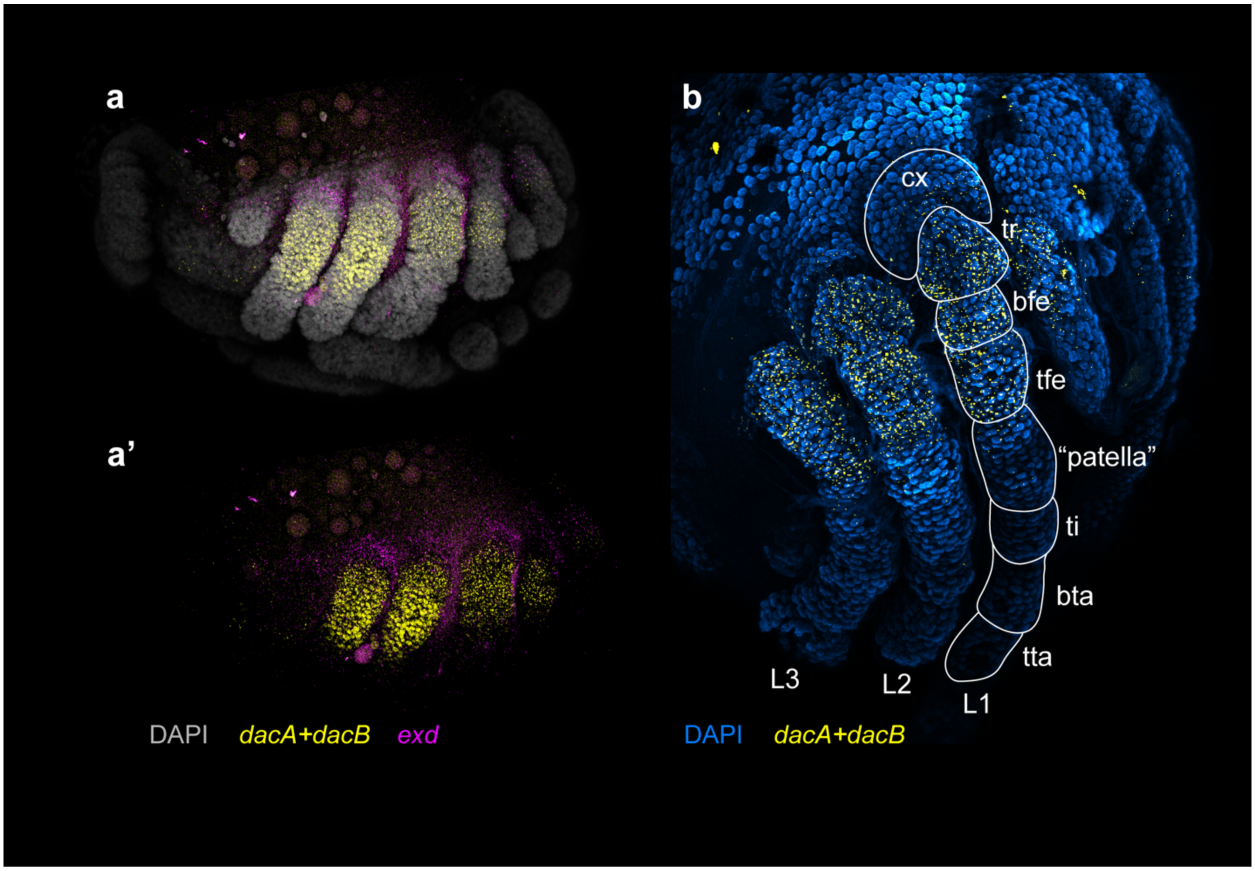

Table S1. Select alternative terminologies for pedipalp podomeres across chelicerate orders. Abbreviations: Cx – coxa; Tr – trochanter; Fe – femur; Tita – tibiotarsus; Pa – patella; Ti – tibia; Ta – tarsus; Mt – metatarsus; Bta – basitarsus; Dta – distitarsus; Gn – genu.

Order	P-I	P-II	P-III	P-IV	P-V	P-VI	Terminus	Reference
Scorpiones	Cx	Tr	Pre-Fe	Fe	Tita	Movable Finger (Chela)	——	Millot & Vachon (1949a)
Scorpiones	Cx	Tr	Fe	Pa	Ti	Ta	——	Hjelle (1990)
Pseudoscorpiones	Cx	Tr	Fe	Ti	Chela	Movable Finger	——	Chamberlin (1931)
Pseudoscorpiones	Cx	Tr	Fe	Ti	Mt	Ta	——	Savory (1964)
Pseudoscorpiones	Cx	Tr	Pre-Fe	Fe	Tita	Pretarsus	——	Weygoldt (1969)
Pseudoscorpiones	Cx	Tr	Fe	Pa	Ti (Chela)	Ta (Movable Finger)	——	Shultz (1989); Harvey (1992)
Araneae	Cx	Tr	Fe	Pa	Ti	Ta	Claws	Foelix (2011)
Amblypygi	Cx	Tr	Fe	Ti	Bta	Dta	Claws	Millot (1949a); Weygoldt (2000)
Amblypygi	Cx	Tr	Fe	Pa	Ti	Ta	Post-Tarsus	Savory (1964)
Uropygi	Cx	Tr	Fe	Ti	Bta	Ta	——	Graveley (1915); Savory (1964); Millot (1949d)
Uropygi	Cx	Tr	Fe	Pa	Ti	Ta	Apotele	Shultz (1993)
Schizomida	Cx	Tr	Fe	Ti	Bta	Ta	Epitarsus	Millot (1949d); Savory (1964)
Schizomida	Cx	Tr	Fe	Pa	Ti	Ta	Spur	Rowland (1975)
Ricinulei	Cx	Tr 1	Tr 2	Fe	Ti	Ta	Spur (Éperon)	Millot (1949c); Pittard & Mitchell (1972)
Palpigradi	Cx	Tr	Fe	Ti	Bta	Ta	Pseudonychium	Millot (1949b)
Palpigradi	Cx	Tr	Fe	Ti	Mt	Ta	——	Börner (1901)
Palpigradi	Cx	Tr	Fe	Gn	Ti	Ta	Apotele	van der Hammen (1982)
Solifugae	Cx	Tr	Fe	Ti	Bta	Ta	Adhesive Organ	Millot & Vachon (1949b)
Solifugae	Cx	Tr	Fe	Ti	Mt	Ta	Adhesive Organ	Punzo (1998)
Opiliones	Cx	Tr	Fe	Pa	Ti	Ta	Claws	Shultz & Pinto-da-Rocha (2007)
Parasitiformes	Cx	Tr	Fe	Gn	Ti	Ta	Apotele	van der Hammen (1966);

								Krantz & Walter (2009)

Table S2. Select alternative terminologies for walking leg podomeres across chelicerate orders. Abbreviations: Cx – coxa; Fe – femur; Ti – tibia; Ta – tarsus; Pr – propodus; Tr – trochanter; Pa – patella; Tita – tibiotarsus; Bta – basitarsus; Tta – telotarsus; Bfe – basifemur; Tfe – telofemur; Mt – metatarsus; Gn – genu; Bti – basitibia; Tti – telotibia; Ata – acrotarsus; Btr – basitrochanter; Ttr – telotrochanter.

Order	P-I	P-II	P-III	P-IV	P-V	P-VI	P-VII	P-VIII	P-IX	Terminus	Reference
Pantopoda (Pycnogonida)	Cx 1	Cx 2	Cx 3	Fe	Ti 1	Ti 2	Ta	Pr	——	Main Claw	Sars (1891); Helfer & Schlottke; King (1973)
Pantopoda	Cx 1	Cx 2	Cx 3	Fe	Ti 1	Ti 2	Ta 1	Ta 2	——	Main Claw	Meinert (1899)
Pantopoda	Cx	Tr 1	Tr 2	Fe	Pa	Ti	Ta 1	Ta 2	——	Pretarsus	Snodgrass (1958); Schram & Hedgpeth (1978)
Xiphosura (L1-4)	Cx	Tr	Fe	Pa	Tita	Pre-tarsus (movable finger)	——	——	——	——	van der Hammen (1989); Shultz (1989)
Xiphosura (L5)	Cx	Tr	Fe	Pa	Ti	Ta	Pre-tarsus	——	——	——	Shultz (1989)
Scorpiones	Cx	Tr	Fe	Ti	Ta 1	Ta 2	Ta 3	——	——	——	Kraepelin (1891)
Scorpiones	Cx	Trans-Cx	Pre-Fe	Fe	Ti	Bta	Ta	——	——	——	Millot & Vachon (1949a)
Scorpiones	Cx	Tr	Fe	Pa	Ti	Bta	Tta	——	——	——	Hjelle (1990)
Pseudoscorpiones	Cx	Tr	Bfe	Tfe	Ti	Mt	Ta	——	——	Arolium & Claws	Chamberlin (1931)
Pseudoscorpiones	Cx	Tr	Bfe	Tfe	Ti	Mt	Ta	——	——	Pretarsus	Savory (1964)
Pseudoscorpiones	Cx	Tr	Fe	Pa	Ti	Mt	Ta	——	——	Arolium & Claws	Shultz (1989); Harvey (1992)
Pseudoscorpiones (Monosphyronida)	Cx	Tr	Fe	Pa	Ti	Mio-Ta	——	——	——	Arolium & Claws	Chamberlin (1931)
Pseudoscorpiones (Feaelloidea & Elassommatina)	Cx	Tr	Fe	Pa	Ti	Ta	——	——	——	Arolium & Claws	Harvey (1992)
Araneae	Cx	Tr	Fe	Pa	Ti	Mt	Ta	——	——	——	Foelix (2011)
Amblypygi	Cx	Tr	Fe	Pa	Ti	Bta	Tta	——	——	Apotele	Millot (1949a); Weygoldt (2000)
Uropygi	Cx	Tr	Fe	Pa	Ti	Bta	Tta	——	——	——	Millot (1949d)
Uropygi	Cx	Tr	Fe	Pa	Ti	Mt	Ta	——	——	——	Savory (1964)
Schizomida	Cx	Tr	Fe	Pa	Ti	Bta	Ta	——	——	——	Millot (1949d); Rowland (1975)
Schizomida	Cx	Tr	Fe	Pa	Ti	Mt	Ta	——	——	——	Savory (1964)
Ricinulei (L1-2)	Cx	Tr	Fe	Pa	Ti	Bta	Ta	——	——	Claws	Millot (1949c)

Ricinulei (L1-2)	Cx	Tr	Fe	Pa	Ti	Mt	Ta	——	——	Claws	Pittard & Mitchell (1972)
Ricinulei (L3-4)	Cx	Tr 1	Tr 2	Fe	Pa	Bta	Ta	——	——	Claws	Millot (1949c)
Ricinulei (L3-4)	Cx	Tr 1	Tr 2	Fe	Pa	Mt	Ta	——	——	Claws	Pittard & Mitchell (1972)
Palpigradi	Cx	Tr	Fe	Pa	Ti	Mt	Ta		——	——	Börner (1901)
Palpigradi	Cx	Tr	Fe	Pa	Ti	Bta	Ta	——	——	Pretarsus	Shultz (1989)
Palpigradi	Cx	Tr	Fe 1	Fe 2	Gn	Ti	Ta	——	——	Apotele	van der Hammen (1982)
Solifugae (L1-2)	Cx	Tr	Pre-Fe	Post-Fe	Ti	Bta	Ta	——	——	——	Millot & Vachon (1949b); Punzo (1998)
Solifugae (L1-L2)	Cx	Tr	Fe	Pa	Ti	Bta	Ta	——	——	——	Shultz (1989)
Solifugae (L3-4)	Cx	Tr 1	Tr 2	Pre-Fe	Post-Fe	Ti	Bta	Ta	——	——	Millot & Vachon (1949b); Punzo (1998)
Solifugae (L3-4)	Cx	Tr	Bfe	Tfe	Pa	Ti	Bta	Ta	——	——	Shultz (1989)
Opiliones	Cx	Tr	Fe	Pa	Ti	Mt	Ta	——	——	Claws	Shultz & Pinto-da-Rocha (2007)
Parasitiformes (Opilioacarida, L1)	Cx	Tr	Bfe	Tfe	Gn	Bti	Tti	Bta	Tta	Apotele	van der Hammen (1966)
Parasitiformes (Opilioacarida, L2)	Cx	Tr	Fe	Gn	Ti	Bta	Tta	Ata	——	Apotele	van der Hammen (1966)
Parasitiformes (Opilioacarida, L3-L4)	Cx	Tr 1	Tr 2	Fe	Gn	Ti	Bta	Tta	Ata	Apotele	van der Hammen (1966)
Parasitiformes (Opilioacarida, L1)	Cx	Tr	Bfe	Tfe	Gn	Ti	Bta	Tta	——	——	Krantz & Walter (2009)
Parasitiformes (Opilioacarida, L2)	Cx	Tr	Fe	Gn	Ti	Bta	Tta	——	——	——	Krantz & Walter (2009)
Parasitiformes (Opilioacarida, L3-4)	Cx	Btr	Ttr	Fe	Gn	Ti	Bta	Tta	——	——	Krantz & Walter (2009)
Parasitiformes (Holothyrida)	Cx	Tr	Bfe	Tfe	Gn	Ti	Ta	——	——	Ambulacrum & Claws	Krantz & Walter (2009)
Parasitiformes (Ixodida)	Cx	Tr	Bfe	Tfe	Gn	Ti	Bta	Tta	——	Claws	Krantz & Walter (2009)
Parasitiformes (Mesostigmata)	Cx	Tr	Bfe	Tfe	Gn	Ti	Bta	Tta	——	——	Krantz & Walter (2009)
Acariformes (Trombidiformes)	Cx	L-1	L-2	L-3	L-4	L-5	L-6	——	——	——	Cook (1974)
Acariformes (Trombidiformes, Endeostigmata, Parasitengona)	Cx	Tr	Bfe	Tfe	Gn	Ti	Ta	——	——	Apotele	Evans (1992)
Acariformes (Oribatida)	Cx	Tr	Fe	Gn	Ti	Ta	——	——	——	Apotele	Evans (1992); Krantz & Walter (2009)

Supplementary Methods

Hybridization Chain Reaction Probe Design

Probes for *Archegozetes longisetosus exd* (17 probe pairs) and *Pycnogonum litorale Dll* (19 probe pairs) were designed by Molecular Instruments using the following CDS templates:

>*Alon_exd*
ATGGACGATAATCAACACAATCATTCACTATCAATAATGCATGCGGTCTCTCAACAAATGTCGGGTCATGTAGTGC
CACAACCGCCTCACGGATACGGGCTGTCCGCTGGTCACGACCCGACCGGTACCACCAATTCCGATGGAGAAACAC
GAAAACATGACATCTCAGAGATATTACAGCAAATCATGAATATTACTGACCAAAGTCTCGATGAGGCTCAGGCCA
GGTCTGTATTGACAACAAATAACAGGAAACATACTTTGAATTGTCATCGCATGAAGCCAGCACTATTCAGCGTTTT
ATGTGAAATCAAGGAAAAGACAGTATTGAGCTTAAGAAACACTCAAGAAGAAGAACCACCAGATCCACAACTGA
TGCGGTTAGACAATATGCTAATAGCGGAAGGGGTTGCAGGACCCGAAAAAGGTGGCGGTCAGTCTGCGGCCGCTA
ATGCGGCGGCAGCTGCGGCTGCGGGTCAGTCGGGAGGTGAAAATGCAATCGAGCACTCAGACTATAGGGCCAAAC
TCGCACAAATTAGAACAATATACCATCAAGAACTTGAGAAATATGAACAGGCCTGTAACGAGTTCACAACACATG
TTATGAATCTATTACGAGAGCAAAGTCGGACGCGTCCAATTACGCCAAAAGAAATCGAAAGAATGGTTCAAATTA
TACACAAAAAGTTTAATTCAATTCAAGTACAGCTCAAACAAAGCACCTGTGAAGCAGTTATGATTCTTAGATCAAG
GTTTTTGGATGCCAGGAGAAAACGAAGAAATTTCAGCAAGCAAGCAACTGAAATATTAAATGAATATTTTTATTCA
CACCTGAGTAACCCATACCCGAGTGAAGAAGCAAAAGAAGAATTAGCGCGAAAGTGTAGTATTACAGTATCTCAA
GTATCAAATTGGTTCGGAAATAAGAGAATTAGGTATAAGAAAAATATTGGAAAAGCTCAAGAAGAAGCAAATCTT
TATGCAGCTAAAAAAGCAGCCGGTTCGTCCCCATACGGTTTAACGCCATCATCTCAGGGCTCGGGAAATATAATGA
GTCCACCGCCACCACCTGGTAGTTCACAGGATGGATGGTCTATGAATGGTGACTATGGAAGTCAGGCCCATAGGC
ACGGAGGCTATTCCTCAGATGGTTCCTCGGGAGGCATGTATGATCCCGGGATGCATCAGGTATCACCTCTTTGA

>*Plit_Dll*
AGTTTCAAAAAATTATTTTTTATATGTTTACGGTGAAGCGTTGAATTTTGTTTTTTTTTAATTTAAAAAAAAAAATCTT
ATTTTATTATTTGACTTTGTTCGGTGTAAACTAACGGCCGTCACCACGTTGAATTTTACGGATTTGTCGTGTCCATA
CCCGATAGCCACTAGTATGGCGGGTAATTCGGATCACGGTTCAATCGAACAAGAACATTTGTCCAAACAGTCCGCA
TTCATGGAGATTCAACAAGCTGGCGGACTGGGACCTCATCACGGGACTACGACGTATCCTATCAGGTCAAGTTATC
AGAGTCAACCGCCTCAACATCACGATAGTGCGTTCGCCATGTCTCATGCTCAACAAGCTGCGGCGGCGGCCGCCG
GTCGTTCAGCCTTAGGACCGTATCATTTCCCGATGAATGCGCATAATACTCCCGCTGGTTATCCAACTGCTCATCCT
TATCTCGGAGCGTATCAGACATCGTGTCCTTCACCTCCAAGAGACGATAAATCGCACATGGAAGAAACATTACGA
GTAAATGGAAAAGGTAAAAAAATGCGTAAACCTAGAACAATATACTCGAGTTTACAGCTACAACAGTTGAATAGA
AGATTTCAAAGAACCCAGTATTTAGCTTTACCCGAAAGAGCCGAATTAGCAGCTTCATTAGGACTTACACAAACAC
AGGTAAAAATATGGTTTCAAAATAGACGGTCGAAATACAAAAAGCTGATGAAGAGTAATCCAACACCTGGTGGAC
CGATTCCACACTTAGGTGGACCGCCATCGCAGCACAGTGTCAATAACGGACCTCTCCAGCATCCGAATCCGGCTAC
AACTCCACAAACTCCACCGTCGCAACAAAATCATCCGTCACATAGTCCACATAGTGATCATCATCATCATCATCCA
TTAGGTAATAACGGTCCTCACGGGCTTGGATTGTTACCTGTTTCGTGCGGACCCGGTATAAGTCCGTCTTCGATAAG
TTCTCCGATGGACTCTTGGAGTATGAACAGTGTTGCCGCCAAAGCCGCTGCTGCAGCCGCGGCAGTTAACGTCACC
AATTCTTACATGAGTCAATATCCTTGGTATCATCAGCCTGATCCGAGTATTAATCAACAATTATTAACGTAG

Table S3. Probe pairs designed for *Phalangium opilio exd* HCR in situ hybridization (B2 initiator).

Pair	Initiator	Spacer	Hybridization	Hybridization	Spacer	Initiator
1	CCTCGTAAATCC TCATCA	AA	TATACCCGGGTCGTACATT CCTGCC	TTACGGATGCAATTCCGGT GGCTGA	AA	ATCATCCAGTAA ACCGCC
2	CCTCGTAAATCC TCATCA	AA	TCCTCCATCTGAGAAATGA CGTGCC	GGCTGAGGCGGCATCCCG TCAGAAT	AA	ATCATCCAGTAA ACCGCC
3	CCTCGTAAATCC TCATCA	AA	CGGCGTTGTCCATAGTGGA CGTAAC	GCGCATTCCCTTGCGTTTG GACAAG	AA	ATCATCCAGTAA ACCGCC
4	CCTCGTAAATCC TCATCA	AA	GACTTGACCATTCGAAATG GCGAAC	AGGAACGCTCGTGTGATA GTTATCG	AA	ATCATCCAGTAA ACCGCC
5	CCTCGTAAATCC TCATCA	AA	GAACTGACCATTGGTGGA CACGGAT	CTGCCATCCGTGGATACCG TTGGAG	AA	ATCATCCAGTAA ACCGCC
6	CCTCGTAAATCC TCATCA	AA	TCGTTAGACCAGCCGAAG CGGCTTT	GCGTCGTGGGAAGGCTGT ACTCTGG	AA	ATCATCCAGTAA ACCGCC

7	CCTCGTAAATCC TCATCA	AA	AGCTTTACCAATATTTTTC TTGTAT	GGCCGCGTATAAATTGGCT TCTTCC	AA	ATCATCCAGTAA ACCGCC
8	CCTCGTAAATCC TCATCA	AA	ACCTGGGAGACTGTTATGC CACACT	ATACGTTTGTTACCGAACC AATTAG	AA	ATCATCCAGTAA ACCGCC
9	CCTCGTAAATCC TCATCA	AA	TAGGGTAAGGGTTACTTAA ATGTGA	TGGCTAATTCCTCCTTGGC TTCTTC	AA	ATCATCCAGTAA ACCGCC
10	CCTCGTAAATCC TCATCA	AA	TTGCTTGCTAAAGTTCCGC CTTTTC	GAAGTACTCGTTGAGAATT TCCGTC	AA	ATCATCCAGTAA ACCGCC
11	CCTCGTAAATCC TCATCA	AA	ATCATGACGGCTTCGCACG TGCTCT	CTCGCGTCTAGAAACCTGG ATCGCA	AA	ATCATCCAGTAA ACCGCC
12	CCTCGTAAATCC TCATCA	AA	TCTTGTGGATGATCTGAAC CATTCG	TGAGCTGCACTTGGATGGA GTTGAA	AA	ATCATCCAGTAA ACCGCC
13	CCTCGTAAATCC TCATCA	AA	CCTGCTCTGTTCCCGGAGC AGGTTC	GATCTCCTTGGGCGTGATG GGACGC	AA	ATCATCCAGTAA ACCGCC
14	CCTCGTAAATCC TCATCA	AA	TGTTCGTATTTTTCGAGCT CCTGGT	ACGTGCGTCGTGAACTCGT TGCACG	AA	ATCATCCAGTAA ACCGCC
15	CCTCGTAAATCC TCATCA	AA	CTCTGTAGTCGGAGTGTTC AATGGC	AGATTTGCCTAATCTGCGC CAGTTT	AA	ATCATCCAGTAA ACCGCC
16	CCTCGTAAATCC TCATCA	AA	TGCCGCCGAAGCGTTGGCC GCCGCG	TTCGGGTTGACCGGGCCCG CCCGCC	AA	ATCATCCAGTAA ACCGCC
17	CCTCGTAAATCC TCATCA	AA	ACTCCTTCCGCTATTAACA TATTGT	GGTCCGCTTCCCTTTTCTG GACCGG	AA	ATCATCCAGTAA ACCGCC
18	CCTCGTAAATCC TCATCA	AA	CTTCTTCTTGTGTGTTTCTC AAACT	GCCTCATTAACTGAGGGTC TGGCGG	AA	ATCATCCAGTAA ACCGCC

Table S4. Probe pairs designed for *Phalangium opilio dac* HCR in situ hybridization (B1 initiator).

Pair	Initiator	Spacer	Hybridization	Hybridization	Spacer	Initiator
1	GAGGAGGGCAGC AAACGG	AA	TGGGTTGTCGCTTAAGTC GCCTTGG	CTCGTTATTCGCCGTACTC GTTACG	TA	GAAGAGTCTTCC TTTACG
2	GAGGAGGGCAGC AAACGG	AA	TCATTGTCCTCGTCATCAG CGCACT	CGTTCATCCTCTTCTTCATC TTCCG	TA	GAAGAGTCTTCC TTTACG
3	GAGGAGGGCAGC AAACGG	AA	CGCCCGTGGCGTTACCAT TTCCGGC	TGTAATTGTGTTCGCTGCC GCTGTG	TA	GAAGAGTCTTCC TTTACG
4	GAGGAGGGCAGC AAACGG	AA	TCGGGTACTGTTCTGCGAT AGGTTA	CAAGCCATTACCCGGACCG CCAACG	TA	GAAGAGTCTTCC TTTACG
5	GAGGAGGGCAGC AAACGG	AA	CCTGCAAGAAGCCAGAAG TAGCCAT	ACCGGACTGTGACCGTTGG CCGAGG	TA	GAAGAGTCTTCC TTTACG
6	GAGGAGGGCAGC AAACGG	AA	TGTCAAAACCGTAAAATT TATCCTT	AGAACGTGGGGTCCTTCAG TCGGTG	TA	GAAGAGTCTTCC TTTACG
7	GAGGAGGGCAGC AAACGG	AA	GACCCCGTCCGTATGTCCT TGTGAC	GTCCGCTAGCTCTTCTCTT GATGAG	TA	GAAGAGTCTTCC TTTACG
8	GAGGAGGGCAGC AAACGG	AA	GAGGATGGGTTTATGTTG TTGGAAC	TCTTTAATGACTGAACCGT CCGTCC	TA	GAAGAGTCTTCC TTTACG
9	GAGGAGGGCAGC AAACGG	AA	AGACGAGGAAGATGAGG ATGATGAT	AGCCGCCGCCTGGGCGGCC GCCGAT	TA	GAAGAGTCTTCC TTTACG
10	GAGGAGGGCAGC AAACGG	AA	CATGAAAGGCAGCGGGTT GATGTGA	CCCGTGGGCGTTGGGGTGG TTCAGC	TA	GAAGAGTCTTCC TTTACG
11	GAGGAGGGCAGC AAACGG	AA	AGCCGTTCGGTAACAAAC CGTGCTT	CCGCTTGGGCCGCCGCGTG GTGACT	TA	GAAGAGTCTTCC TTTACG
12	GAGGAGGGCAGC AAACGG	AA	TCGGCTTTTGGAAATGTGT CCGTTT	CAATCGTTCACCTGTTGAA TAGAAA	TA	GAAGAGTCTTCC TTTACG
13	GAGGAGGGCAGC AAACGG	AA	CCTTTCTTGAGAAGGCCG GCGTGTG	TATCCACCGTACTCTCCGT CAATTC	TA	GAAGAGTCTTCC TTTACG
14	GAGGAGGGCAGC AAACGG	AA	ACGCTCTCTTTGGTGGTCG GCCCGG	TGGAGGAACTGGTCAGTCC CACCAT	TA	GAAGAGTCTTCC TTTACG
15	GAGGAGGGCAGC AAACGG	AA	TGTGCAATCTTTATACAA GGAGTCG	GCCAGAAGAGAAGACACA CCTCGCT	TA	GAAGAGTCTTCC TTTACG
16	GAGGAGGGCAGC AAACGG	AA	TTGACTCCGGGTTGTATG GCTCCTA	TCTTTGCAGCTGAGTAATT TGCAAC	TA	GAAGAGTCTTCC TTTACG
17	GAGGAGGGCAGC AAACGG	AA	TACAAACGATGGGAGTTA TGTCCAA	CTCTGAGAATTCGAACTTG TTCGAC	TA	GAAGAGTCTTCC TTTACG
18	GAGGAGGGCAGC AAACGG	AA	TCCTCCGACCAGGTGTTTC AAAAAC	TTTGAGTTTAGTGTAGACC GTGTGC	TA	GAAGAGTCTTCC TTTACG
19	GAGGAGGGCAGC AAACGG	AA	TATTCTCCACCGACCAAG AAAGCGG	TCGAAAGCTTGGGGTAGAC AAAGTA	TA	GAAGAGTCTTCC TTTACG
20	GAGGAGGGCAGC AAACGG	AA	GGCATTCGTTGCCGACGC CGTCACC	CTTTAGCTCCGCGATAATC TATCAA	TA	GAAGAGTCTTCC TTTACG

Table S5. Probe pairs designed for *Phalangium opilio Notch* HCR in situ hybridization (B3 initiator).

Pair	Initiator	Spacer	Hybridization	Hybridization	Spacer	Initiator
1	GTCCCTGCCTCTATATCT	TT	ATGGGCGTTTTGAGATTGCTGATTT	TCAAATGAAAACCGCTTCTTGGGGT	TT	CCACTCAACTTTAACCCG
2	GTCCCTGCCTCTATATCT	TT	GTCCGATTGAGCGGAATGGGGTGAA	CAAAGGGCTAGAGATTCCTTCCGAC	TT	CCACTCAACTTTAACCCG
3	GTCCCTGCCTCTATATCT	TT	AACCACCGTGGCTATGTTGGGAAGG	GTAAATAATGTTGAGGGGTAGCTTC	TT	CCACTCAACTTTAACCCG
4	GTCCCTGCCTCTATATCT	TT	CCACCACTCAAGAGCATTTGAAGGT	TGTCCACCCATATTGTTTCCGCCAC	TT	CCACTCAACTTTAACCCG
5	GTCCCTGCCTCTATATCT	TT	GAAGTTGGCAATGATGGTCGTTGTT	GCCCTCATAACGGCCATGTGAGTGG	TT	CCACTCAACTTTAACCCG
6	GTCCCTGCCTCTATATCT	TT	GTTTTGACGAGGGTGATGCGCTTTC	TCCGTTACTCATACCACCAGGCATT	TT	CCACTCAACTTTAACCCG
7	GTCCCTGCCTCTATATCT	TT	CACCCATCCCGGTTAAATGGTCCAT	CCCCTAAATACATTTGGCCTCCGTT	TT	CCACTCAACTTTAACCCG
8	GTCCCTGCCTCTATATCT	TT	AGGTCCAAGTTGGCCAAATTGGGGT	CCCATTTGCTGTTGGCAAGCGTTAA	TT	CCACTCAACTTTAACCCG
9	GTCCCTGCCTCTATATCT	TT	TAGAGAATTAACTGGAGAGAGCGTC	ATCGACGCAACCCATACCAGGGGAT	TT	CCACTCAACTTTAACCCG
10	GTCCCTGCCTCTATATCT	TT	CACCGCCTTCATTGACCGGCATCAT	TGGATGGTTTCCTCCTGGTACTACC	TT	CCACTCAACTTTAACCCG
11	GTCCCTGCCTCTATATCT	TT	TTAGACATCTGCTGACTCGGTGGCA	GGGGAACCGATAGTAGCGTTAGACA	TT	CCACTCAACTTTAACCCG
12	GTCCCTGCCTCTATATCT	TT	ATCCCGATTTGCGTAATTTTCAAGA	CGGTAACCGATCCATATGATCAGTT	TT	CCACTCAACTTTAACCCG
13	GTCCCTGCCTCTATATCT	TT	CTCCGTGGCTAAGTAGAACCCTGAC	CTTTATTGTCTTGAGCATCTCTGTT	TT	CCACTCAACTTTAACCCG
14	GTCCCTGCCTCTATATCT	TT	ATGAGATCTTCCACCATGCCCTCAA	GCCGCATTGATGTCAGCTTCAGCAT	TT	CCACTCAACTTTAACCCG
15	GTCCCTGCCTCTATATCT	TT	CTGAAAAACGCCTTGGGCATCGGCA	GTTGGTGGCACGATTTCGAAGCAAA	TT	CCACTCAACTTTAACCCG
16	GTCCCTGCCTCTATATCT	TT	AAGATTCTCCAGTTCGCTCGGTAGT	TGGCATAACGCGCGGCTAAGTGAAG	TT	CCACTCAACTTTAACCCG
17	GTCCCTGCCTCTATATCT	TT	ACCGCCTCGGAAAGACGCCAACATT	TTCTTCGTCTTCGCCAGTGTCCAAA	TT	CCACTCAACTTTAACCCG
18	GTCCCTGCCTCTATATCT	TT	TCCACGTTGCCATGATCGAGATCAC	GGAGTAAAACCGGCTGGTCCTTGTA	TT	CCACTCAACTTTAACCCG
19	GTCCCTGCCTCTATATCT	TT	GTTCCTAACATCGGCGGCGTCCAAA	AGGAGGAGTCAAGGCCAAAATGTCT	TT	CCACTCAACTTTAACCCG
20	GTCCCTGCCTCTATATCT	TT	TCGTCATAGTCGGCGGTAGTCATGA	TGTTGAGTCCATGGTCGAGGATCGT	TT	CCACTCAACTTTAACCCG

Table S6. Probe pairs designed for *Phalangium opilio engrailed* HCR in situ hybridization (B1 initiator).

Pair	Initiator	Spacer	Hybridization	Hybridization	Spacer	Initiator
1	GAGGAGGGCAGCAAACGG	AA	ACCTTCGCCATCCATGGCGACGGTA	CTATGATTTGTCCATTGAATCATCT	AA	GAAGAGTCTTCCTTTACG
2	GAGGAGGGCAGCAAACGG	AA	TCTGAAACCAAATTTTAATTTGGGA	TGGCCTTCTTGATTTTGGCCCGTTT	AA	GAAGAGTCTTCCTTTACG
3	GAGGAGGGCAGCAAACGG	AA	GAACTCTTGTTTTAGCCGGGCCAAC	TTTTTCCGTCAAGTATCTGTTTTCC	AA	GAAGAGTCTTCCTTTACG
4	GAGGAGGGCAGCAAACGG	AA	TTCTCATCAGGTTTCTTTTCCTTCT	TCGGCGGTGAACGCTGTCCTGGGTC	AA	GAAGAGTCTTCCTTTACG
5	GAGGAGGGCAGCAAACGG	AA	CAGATGAGGGTCTGTCGGAGTATCG	TCATGCGTCGGGACCGGGGACCTGG	AA	GAAGAGTCTTCCTTTACG
6	GAGGAGGGCAGCAAACGG	AA	GGGTCCCTTTTTGCCCGTTTCGCCG	GCAATAGACCCAGGCGGGCCATAAA	AA	GAAGAGTCTTCCTTTACG
7	GAGGAGGGCAGCAAACGG	AA	GGCGACTGGCTGGTTTCGGGCGTGG	TCTTGGGGCGCCAAGGAGGGCAATT	AA	GAAGAGTCTTCCTTTACG
8	GAGGAGGGCAGCAAACGG	AA	AGTTAACGGAACTACAGTTCGAACT	CGCCGACGCTCCGAGGCCTAACGGA	AA	GAAGAGTCTTCCTTTACG
9	GAGGAGGGCAGCAAACGG	AA	ATGATTGCGATGGTGTTCGCGATTG	ACTTTCGGGTTGTTGAACGCGCCGG	AA	GAAGAGTCTTCCTTTACG
10	GAGGAGGGCAGCAAACGG	AA	ACCGCCGAGAAAAAGTCCGTCGGAG	TTGCTGGCGGGTGTTGGCGGACCGG	AA	GAAGAGTCTTCCTTTACG
11	GAGGAGGGCAGCAAACGG	AA	TATGAACGAGTCCGTTATGAGTCCT	GGGGCACGGCCGCGTGATGTAGGTG	AA	GAAGAGTCTTCCTTTACG

12	GAGGAGGGCAGCAAACGG	AA	TTTAGGAGTTTTGCCGAATTCCGGC	GACTTCGGCCGCGATGGGCGACCCG	AA	GAAGAGTCTTCCTTTACG
13	GAGGAGGGCAGCAAACGG	AA	GAGTGCCTGGAAAAGGGCGCCCCAA	AAAATCTTCTCGATGGAAAACTTGA	AA	GAAGAGTCTTCCTTTACG
14	GAGGAGGGCAGCAAACGG	AA	AGTTCTCTACGGCGCCGGGGAAGGA	CGGCTGGTATGACCGGAGGCGCCCG	AA	GAAGAGTCTTCCTTTACG
15	GAGGAGGGCAGCAAACGG	AA	ATCGTCGACGCGGGAACTGTCCGCG	ATTTCGCTGAGGAGGCGGCGTCGTT	AA	GAAGAGTCTTCCTTTACG
16	GAGGAGGGCAGCAAACGG	AA	CGCGCCGGGCTGCTGATCGACGTTC	GACGACGTACAGTCCAAGTCGTCGG	AA	GAAGAGTCTTCCTTTACG
17	GAGGAGGGCAGCAAACGG	AA	CCATAGCGCCCGCTTTTCGAGACCG	GCTTGGCCTCTACCATCGTATCTAA	AA	GAAGAGTCTTCCTTTACG
18	GAGGAGGGCAGCAAACGG	AA	CGACTACGGTGGTCTTACCCGAAGA	GGGGTCCGATCTTTCTCCCCGCCGC	AA	GAAGAGTCTTCCTTTACG
19	GAGGAGGGCAGCAAACGG	AA	TTCTCTCTCGTAATAAGTAAAACTC	ACTCGAACCTCGAGTAAGATTGAAA	AA	GAAGAGTCTTCCTTTACG

Table S7. Probe pairs designed for *Phalangium opilio Distal-less* HCR in situ hybridization (B3 initiator).

Pair	Initiator	Spacer	Hybridization	Hybridization	Spacer	Initiator
1	GTCCCTGCCTCTATATCT	TT	TCGAAGAACTCCAGCGATTGATTAT	TTTTTCCGATAGGTTTTCGGGATGA	TT	CCACTCAACTTTAACCCG
2	GTCCCTGCCTCTATATCT	TT	CCCGTGTGATGGTTGCGCATTTGTC	CATATGCACGATGTTCACGTGTTCA	TT	CCACTCAACTTTAACCCG
3	GTCCCTGCCTCTATATCT	TT	TCTGGGAACGGTAATAGCTCGTTCC	CCCGGTTTGGTTTACTTCTTGTCGC	TT	CCACTCAACTTTAACCCG
4	GTCCCTGCCTCTATATCT	TT	ATTCGTGAGTGATTGGTTTTTGTTT	GAACTCGAAGTAACGCGAAGAGGGT	TT	CCACTCAACTTTAACCCG
5	GTCCCTGCCTCTATATCT	TT	TTCACGGGGTTATGTTAAAATTTGG	AATATAAACCACGAGACGTGACGTA	TT	CCACTCAACTTTAACCCG
6	GTCCCTGCCTCTATATCT	TT	CAAGAATACTGTGGCATGTAACCGT	GTTAACGAAGGGTCCGCTTGGTGAT	TT	CCACTCAACTTTAACCCG
7	GTCCCTGCCTCTATATCT	TT	GATTCATGTCCCAAGAACTAATAGG	TATGCATGTTCATAGCGGCTTTCGC	TT	CCACTCAACTTTAACCCG
8	GTCCCTGCCTCTATATCT	TT	CCCGATTGGGTCATCATGCCCGATT	GCGTTGGACATGCTATTTACGGGCG	TT	CCACTCAACTTTAACCCG
9	GTCCCTGCCTCTATATCT	TT	TCGTTGCCGGGTTTGGTTGCTGTAA	AATGTCCTTCCGACGGAGTTTGAGG	TT	CCACTCAACTTTAACCCG
10	GTCCCTGCCTCTATATCT	TT	CTTGTTGTTGAGCTTTCAACATTTT	GACCGCCCGGGCCCGAATTCGGAGG	TT	CCACTCAACTTTAACCCG
11	GTCCCTGCCTCTATATCT	TT	GATTTTCACCTGCGTTTGCGTTAGA	GTACTTGGAACGGCGATTTTGGAAC	TT	CCACTCAACTTTAACCCG
12	GTCCCTGCCTCTATATCT	TT	GGTAACGCCAAGTACTGCGTTCTTT	AGAGACGCGGCTAATTCCGCTCTTT	TT	CCACTCAACTTTAACCCG
13	GTCCCTGCCTCTATATCT	TT	GACTCGAGTATATCGTTCTGGGCTT	ACCTTCTGTTTAATTGCTGTAATTG	TT	CCACTCAACTTTAACCCG
14	GTCCCTGCCTCTATATCT	TT	GTCTCTTCTAAACCAGATTTGTCGT	TTTTTGCCCTTTCCATTGACTCTCA	TT	CCACTCAACTTTAACCCG
15	GTCCCTGCCTCTATATCT	TT	TGGCGAGGTAATGGCTAGGATGAGT	CACACGAACCGACGTTGGTCGGATA	TT	CCACTCAACTTTAACCCG
16	GTCCCTGCCTCTATATCT	TT	GGAATTGTTCATGGAAAAGGGGTAG	CGAATTATGCAAACCAGAGTTTAAC	TT	CCACTCAACTTTAACCCG
17	GTCCCTGCCTCTATATCT	TT	GAATTTCCATAAAGGCCGACTTGTT	CATTCAGTCCGGCCGCCGCCTGCTG	TT	CCACTCAACTTTAACCCG
18	GTCCCTGCCTCTATATCT	TT	GGTGGCGGCTTTGGCGGAACAACGT	AGCCGAGAACTCGCTAAAATTCAAC	TT	CCACTCAACTTTAACCCG

Table S8. Probe pairs designed for *Pselaphochernes scorpioides exd-1* HCR in situ hybridization (B2 initiator).

Pair	Initiator	Spacer	Hybridization	Hybridization	Spacer	Initiator
1	CCTCGTAAATCCTCATCA	AA	CACCCAAAGCTCTTGATTAAAATAA	GGGGACACTCACGACAAATGCGAGT	AA	ATCATCCAGTAAACCGCC
2	CCTCGTAAATCCTCATCA	AA	CGCCTATCCTCCATAGGCGAGGGAG	TTTTTACAAGAAGTCGAACCCTAAG	AA	ATCATCCAGTAAACCGCC
3	CCTCGTAAATCCTCATCA	AA	CTGGCACCCATGGAGGAATACGAGT	CAGATTTTGTAGTCCTGGGATGGCA	AA	ATCATCCAGTAAACCGCC

4	CCTCGTAAATCC TCATCA	AA	GAGTGGAAACCATGTTATA GGGAGA	GGGGCGGACTAATCATGG GTCCCTG	AA	ATCATCCAGTAA ACCGCC
5	CCTCGTAAATCC TCATCA	AA	CTTCTTCCTGAGCTTTGCC GATATT	CCGCTTTCTTGGCGGCGTA CAGGTT	AA	ATCATCCAGTAA ACCGCC
6	CCTCGTAAATCC TCATCA	AA	CCAATTGGAGACCTGAGAT ACGGTG	CTTGTACCGGATTCTCTTG TTGCCG	AA	ATCATCCAGTAA ACCGCC
7	CCTCGTAAATCC TCATCA	AA	CCTCTTCACTGGGGTACGG ATTGCT	AACACTTCCTAGCCAGCT CTTCCTT	AA	ATCATCCAGTAA ACCGCC
8	CCTCGTAAATCC TCATCA	AA	CTCGGTCGCCTGTTTGCTG AAGTTT	GTGCGAGTAGAAGTACTC GTTGAGG	AA	ATCATCCAGTAA ACCGCC
9	CCTCGTAAATCC TCATCA	AA	ATCTGAGGATCATAACGGC TTCACA	GTTTCCGCCTTGCGTCGAG GAACCT	AA	ATCATCCAGTAA ACCGCC
10	CCTCGTAAATCC TCATCA	AA	GTTGAATTTGCGGTGGATG ATCTGA	GGACTGCTTGAGTTGGAC TTGGATG	AA	ATCATCCAGTAA ACCGCC
11	CCTCGTAAATCC TCATCA	AA	ACTCATTGCAGGCCTGCTC GTACTT	TCAGCAGGTTCATGACGT GGGTGGT	AA	ATCATCCAGTAA ACCGCC
12	CCTCGTAAATCC TCATCA	AA	TCGGGAGGCTCCTCATCTT GTGTGT	ATGTTGTCCAGCCGCATA AGCTGAG	AA	ATCATCCAGTAA ACCGCC
13	CCTCGTAAATCC TCATCA	AA	GAAGAGTGCAGGTTTCATG CGATGG	TTTTTCTTTGATTTCACAT AGAACA	AA	ATCATCCAGTAA ACCGCC
14	CCTCGTAAATCC TCATCA	AA	TCATCCAGACTTTGGTCCG TTATAT	TTGAGCGTATGTTTTCTTG CTTGAG	AA	ATCATCCAGTAA ACCGCC
15	CCTCGTAAATCC TCATCA	AA	GTTCAGAAGGTTGTTGTCC ATTAGG	CTGAAATGTCATGTTTTCT TACATC	AA	ATCATCCAGTAA ACCGCC
16	CCTCGTAAATCC TCATCA	AA	TGCATGCTGAGGCACGAC ATGACCC	AACTTGATGTGGTTGAGG CATGCCA	AA	ATCATCCAGTAA ACCGCC
17	CCTCGTAAATCC TCATCA	AA	GGATGAAGCATTCTTTGTT GCTCGT	ATGCTAACGGCAGGGTGC TGAGTAA	AA	ATCATCCAGTAA ACCGCC
18	CCTCGTAAATCC TCATCA	AA	GGAAGGACATCAGTGATG AAAAACA	TGCTTTAACATCACCCTCG GTGAGC	AA	ATCATCCAGTAA ACCGCC
19	CCTCGTAAATCC TCATCA	AA	AATAAACATCCGCGAGTA CAAAAAC	GCTGGGCAGGTCGTCAGT TGCAAGC	AA	ATCATCCAGTAA ACCGCC
20	CCTCGTAAATCC TCATCA	AA	AAACGTTTGACGGACAAC AAGCAAC	TAATCACCGCAATAAGAG TATTTTG	AA	ATCATCCAGTAA ACCGCC

Table S9. Probe pairs designed for *Pselaphochernes scorpioides exd-2* HCR in situ hybridization (B2 initiator).

Pair	Initiator	Spacer	Hybridization	Hybridization	Spacer	Initiator
1	GTCCCTGCCTCT ATATCT	TT	TCTGGATATGAGGTTCCCT AAGAAG	AATGGTGCTTATATAATGT TTTGTC	TT	CCACTCAACTTT AACCCG
2	GTCCCTGCCTCT ATATCT	TT	ACTTACACTGTACAGAGCG TTTTTC	AACCGATAGAATGATTGTG GTAACA	TT	CCACTCAACTTT AACCCG
3	GTCCCTGCCTCT ATATCT	TT	AATTTCCGCCTGATGGTAG AGTAGG	GGGGATCTCCACAAATAG AAAAATA	TT	CCACTCAACTTT AACCCG
4	GTCCCTGCCTCT ATATCT	TT	TATATACAGTAGATACCAA ATGTCA	GAAAGAGGTGCTCTGCAG TTAGTTG	TT	CCACTCAACTTT AACCCG
5	GTCCCTGCCTCT ATATCT	TT	TCTTGAGCTTTGCCAATGT TCTTTT	TTTTTGGCGGCGTAGAGGT TGGCCT	TT	CCACTCAACTTT AACCCG
6	GTCCCTGCCTCT ATATCT	TT	TGGAGACCTGAGAGACAG TGATGCC	ATCTTATCCTCTTGTTTCCG AACCA	TT	CCACTCAACTTT AACCCG
7	GTCCCTGCCTCT ATATCT	TT	CTCGCTAGGGTAAGGGTTA CTCAAG	CTTCCTCGCCAATTCCTCC TTGGCC	TT	CCACTCAACTTT AACCCG
8	GTCCCTGCCTCT ATATCT	TT	GTCGCCTGCTTGCTGAAGT TGCGCC	GAGTAGAAGTATTCGTTCA GGATCT	TT	CCACTCAACTTT AACCCG
9	GTCCCTGCCTCT ATATCT	TT	TGAGGATCATGACCGCTTC ACACGT	TCCTCCTGGCGTCCAAGAA CCGGGA	TT	CCACTCAACTTT AACCCG
10	GTCCCTGCCTCT ATATCT	TT	GAACTTGCGATGGATGATC TGGACC	CTGCTTGAGTTGAACCTGG ATGGAG	TT	CCACTCAACTTT AACCCG
11	GTCCCTGCCTCT ATATCT	TT	CTCGTCCGGCTCTGCTCCC GTAGTA	CGCTCGATCTCCTTGGGCG TGATGG	TT	CCACTCAACTTT AACCCG
12	GTCCCTGCCTCT ATATCT	TT	AGGCCTGCTCGTATTTCTC GAGTTC	TCATGACGTGGGTGGTGAA CTCGTT	TT	CCACTCAACTTT AACCCG
13	GTCCCTGCCTCT ATATCT	TT	TTTGGCGCGGTAGTCCGAA TGCTCG	GTGATATATAGTACGGATC TGCGCC	TT	CCACTCAACTTT AACCCG
14	GTCCCTGCCTCT ATATCT	TT	GCCGCCGAAGAAACATTC GCTGCAG	GCATTCTCGGCTTGGCCTC CCGACG	TT	CCACTCAACTTT AACCCG
15	GTCCCTGCCTCT ATATCT	TT	CGACGCCCTCGGCTACCAG CATGTT	CGGCACCACCTCCCTTTTC GGGTCC	TT	CCACTCAACTTT AACCCG
16	GTCCCTGCCTCT ATATCT	TT	GAAGAGTGCAGGCTTCATC CGATGG	TTTTTCTTTGATCTCACAG AGAACA	TT	CCACTCAACTTT AACCCG
17	GTCCCTGCCTCT ATATCT	TT	TCATCCAAGCTCTGGTCGG TAATAT	TTGAGGGTATGTTTCCTTG CTTGAG	TT	CCACTCAACTTT AACCCG

18	GTCCCTGCCTCT ATATCT	TT	ACGACGGGGAGGTAACCG CCAGGGT	GGGGTCGCAGTCTCGGGG CTTTGGT	TT	CCACTCAACTTT AACCCG
19	GTCCCTGCCTCT ATATCT	TT	TTTCCATCGCGGGTCACAT CCATAG	GCATACCGGGAGGATGTA ACATATT	TT	CCACTCAACTTT AACCCG
20	GTCCCTGCCTCT ATATCT	TT	TTTCTCAACGCATATAGAA GCCACG	CGCACGCTGGACCGCGGC GACGTAC	TT	CCACTCAACTTT AACCCG

Table S10. Probe pairs designed for *Pselaphochernes scorpioides dac-1* HCR in situ hybridization (B3 initiator).

Pair	Initiator	Spacer	Hybridization	Hybridization	Spacer	Initiator
1	GTCCCTGCCTCTA TATCT	TT	GTTTAATTGGAGGTGGTTT GACATG	TTGGAATCCTACTTAGATG AAATGA	TT	CCACTCAACTTTA ACCCG
2	GTCCCTGCCTCTA TATCT	TT	ACAGGGTTCTCTTATCATC ATCGCG	TAGATAACTTTCACTAATG ATGATG	TT	CCACTCAACTTTA ACCCG
3	GTCCCTGCCTCTA TATCT	TT	TTTCTTTCTCAAAACTTAG CGACTA	CCCCGTATCAAAATATCC CAAGTCA	TT	CCACTCAACTTTA ACCCG
4	GTCCCTGCCTCTA TATCT	TT	CAAAGCGATGCCTCAGAA TGTTTTG	GCGAGCCTTGTCTTCTAAC GAACGA	TT	CCACTCAACTTTA ACCCG
5	GTCCCTGCCTCTA TATCT	TT	ACCAACACGGGATCTTTT ATAAATG	ACCTTCCAACACTTGATG GACCGTA	TT	CCACTCAACTTTA ACCCG
6	GTCCCTGCCTCTA TATCT	TT	GTTTATATTGGATGTGTTA ATGAGG	TCAACGTTTGTTTTTCTCT CGTCTG	TT	CCACTCAACTTTA ACCCG
7	GTCCCTGCCTCTA TATCT	TT	GCCAGAGTGGGTTTTAAA ATGATGG	GAGAATTTTGGGGTGTAT AAAAATG	TT	CCACTCAACTTTA ACCCG
8	GTCCCTGCCTCTA TATCT	TT	CATACAAGACTTGGGAAA CAGGTTT	GAACATCATGTGGTGCCT ATGTATT	TT	CCACTCAACTTTA ACCCG
9	GTCCCTGCCTCTA TATCT	TT	TAGATGTTCAGGCTGACA AAGGACA	TATGGTAGGACTTGATAT GAAGATA	TT	CCACTCAACTTTA ACCCG
10	GTCCCTGCCTCTA TATCT	TT	CCTAGTGTTTCTCTCTCGA TCCAAT	ATCTTGTATTTGGTGCTCT TTTTCA	TT	CCACTCAACTTTA ACCCG
11	GTCCCTGCCTCTA TATCT	TT	AAGGTCTCCGCTGAGGTG TTGCGTA	TGGGCCAAGGAATCATTG AGGAGTC	TT	CCACTCAACTTTA ACCCG
12	GTCCCTGCCTCTA TATCT	TT	TCTCGTTCTCCAGTTGATC CTGGAC	CGTCTTCGTACTGGGAGC GCTTCTT	TT	CCACTCAACTTTA ACCCG
13	GTCCCTGCCTCTA TATCT	TT	CCTTTTCTGGTACACGATT CTTGCT	ACGACGTGATCGCTTTTCT CTCCGG	TT	CCACTCAACTTTA ACCCG
14	GTCCCTGCCTCTA TATCT	TT	CTCTCCCGAACTTCTCGTT CTCTTA	TGTTCGTCCATGAGCTGCT TCTCGA	TT	CCACTCAACTTTA ACCCG
15	GTCCCTGCCTCTA TATCT	TT	GGTTGACCTGTCGTTCTTG CTGTCG	TCTCCATCTTGAGCTCGGC TTTTTC	TT	CCACTCAACTTTA ACCCG
16	GTCCCTGCCTCTA TATCT	TT	TGTTCTCTGTCTTCGTCGT CTTCAT	CTCACGTCGGGGTTGTCG CTCAGCT	TT	CCACTCAACTTTA ACCCG
17	GTCCCTGCCTCTA TATCT	TT	GAACCAACCATAGGTGCC CATTGAT	AGAGATTGAGGACGGGAC TGTGGCC	TT	CCACTCAACTTTA ACCCG
18	GTCCCTGCCTCTA TATCT	TT	TAGCCCGTAGAGACGTTC TTTCTGT	AGCGGTGTCTTTCTGTTTC TGACTC	TT	CCACTCAACTTTA ACCCG
19	GTCCCTGCCTCTA TATCT	TT	GTAAGGAGATCGGCAGAC CGAGCTG	TGGTTTTCCTCCTTTAATC TGGCTG	TT	CCACTCAACTTTA ACCCG
20	GTCCCTGCCTCTA TATCT	TT	GGACTCCGTCCAGGCGGG ACTTCTT	CGTTCTCATAGCTTCCATA GTCAGG	TT	CCACTCAACTTTA ACCCG
21	GTCCCTGCCTCTA TATCT	TT	CAGTCCTTGTACAGGACA TCGAAGT	CTTCCAGGCCTGGAACTT GCCGTAG	TT	CCACTCAACTTTA ACCCG
22	GTCCCTGCCTCTA TATCT	TT	ACGATAGGAGTGATGTCC AATCGCT	AGGATTCGCACCTGCTCG ACGTTGC	TT	CCACTCAACTTTA ACCCG
23	GTCCCTGCCTCTA TATCT	TT	CGACCAGATGTTTCAGGA AGAGTTC	GTTTGGTATAGACAGTGT GAAGACC	TT	CCACTCAACTTTA ACCCG

Table S11. Probe pairs designed for *Pselaphochernes scorpioides dac-2* HCR in situ hybridization (B3 initiator).

Pair	Initiator	Spacer	Hybridization	Hybridization	Spacer	Initiator
1	GTCCCTGCCTCTA TATCT	TT	ATAGAATTGAAATTAAGA GAGTGAG	GGGGTCATCACATATAAA CAGGTAA	TT	CCACTCAACTTTA ACCCG
2	GTCCCTGCCTCTA TATCT	TT	CATCAAGGGCGCTATAAC AATTTGA	TCTACAGAAGAGGCTAAG CTATCAC	TT	CCACTCAACTTTA ACCCG
3	GTCCCTGCCTCTA TATCT	TT	TGCGAAAGGAAGATTTTC CGCGCAG	CCCCACTCCTGGAGAAAT GAAGAAC	TT	CCACTCAACTTTA ACCCG
4	GTCCCTGCCTCTA TATCT	TT	GGCTCGAAGGACTGGGGT TTATGCT	GGGGAATTCTTGATATGTT TTAGAA	TT	CCACTCAACTTTA ACCCG

5	GTCCCTGCCTCTATATCT	TT	GACTCCACTTTCACCATGCGATCAC	ATGGGGCATTCTTGAACTTTATGTT	TT	CCACTCAACTTTAACCCG
6	GTCCCTGCCTCTATATCT	TT	ACCACACGTTGAGGAGTCTCATGGT	CCAGTTCTTGGGTTAATGATTCTGC	TT	CCACTCAACTTTAACCCG
7	GTCCCTGCCTCTATATCT	TT	GTACTGCGCTCTCCTCTTCATTTCA	TGCCGAGCTGTTCCTCAGGGCATCT	TT	CCACTCAACTTTAACCCG
8	GTCCCTGCCTCTATATCT	TT	GTTTTCTTTTCTCGCTTGAATCTCT	TCCAGTTGGTCTTGCACTCGCCGCC	TT	CCACTCAACTTTAACCCG
9	GTCCCTGCCTCTATATCT	TT	CCAGAAGTTGTTTCTCGATGTTTTC	GGTAGAGGATGCGTATTCTTTGTTC	TT	CCACTCAACTTTAACCCG
10	GTCCCTGCCTCTATATCT	TT	CTTTAGCTCGGCTTTATCAATGGAG	CAGTTCCCGTTCTCGCATCATCTCC	TT	CCACTCAACTTTAACCCG
11	GTCCCTGCCTCTATATCT	TT	TTGGCGGATAGGTAAGGTGCAAGAA	AGACCCAAGCCAACATCTCCGGGAG	TT	CCACTCAACTTTAACCCG
12	GTCCCTGCCTCTATATCT	TT	GACATCGGGGTTGTCGCTCAGTTCT	TAAACGATCGGAGTTCGCCGTGGAA	TT	CCACTCAACTTTAACCCG
13	GTCCCTGCCTCTATATCT	TT	GAGTCGTTGTAGTCTTCTTCCGGTG	GGCTCTCGATCATCTTCATCATCTT	TT	CCACTCAACTTTAACCCG
14	GTCCCTGCCTCTATATCT	TT	GCTGCTGTTTTGGGACAAGTTGAGA	GGTGTAGGCTGTGTCTTGATCTCGC	TT	CCACTCAACTTTAACCCG
15	GTCCCTGCCTCTATATCT	TT	TTAAGGTATGCTTGATGTTGCTCCT	GGGCTGTGACCGTTGGTCAGGGTTC	TT	CCACTCAACTTTAACCCG
16	GTCCCTGCCTCTATATCT	TT	GCGCTATGTCTACCAGGTCAGATGA	TCCTTTCGTAGCCGTAATGCCGTTC	TT	CCACTCAACTTTAACCCG
17	GTCCCTGCCTCTATATCT	TT	AGAGTGCTTGATGACGGAGTGTTGA	TGAGGTCTGGAGGCTCAGTTCGGGA	TT	CCACTCAACTTTAACCCG
18	GTCCCTGCCTCTATATCT	TT	TTCCTCCTGTCCCTTCTTCAGCCAA	GGAACCCGTTGGCCACCATTTTGTC	TT	CCACTCAACTTTAACCCG
19	GTCCCTGCCTCTATATCT	TT	CCCAGTTCTGTGATATGTCCGTTCT	TCACGTTTGTACCAGGACGGACGCG	TT	CCACTCAACTTTAACCCG
20	GTCCCTGCCTCTATATCT	TT	CTAGACGGGATTTCTTCAGCAGCTG	ACGAAGTCGTCCCAAAGTCCCCTGC	TT	CCACTCAACTTTAACCCG
21	GTCCCTGCCTCTATATCT	TT	CCTTGTAGAGGCTGTCAAAGTCTTT	GGCGGCCAGGCCTGGCAGTTGTACA	TT	CCACTCAACTTTAACCCG
22	GTCCCTGCCTCTATATCT	TT	CTGACTTGTTCCACGTTACAGACAA	TGGATGGCGCCCAGTCCACGAAGGA	TT	CCACTCAACTTTAACCCG
23	GTCCCTGCCTCTATATCT	TT	TGTAGACTGTGTGGAGGCCTCCCAC	GCATGATATCGAGCCGTTTCAGTTT	TT	CCACTCAACTTTAACCCG
24	GTCCCTGCCTCTATATCT	TT	AGGTAAACAAAGTAGATAATCGCCG	ATGTTTCAGGAAGAGTTCAAAAGCT	TT	CCACTCAACTTTAACCCG

Table S12. Probe pairs designed for *Titanopuga salinarum exd* HCR in situ hybridization (B3 initiator).

Pair	Initiator	Spacer	Hybridization	Hybridization	Spacer	Initiator
1	GTCCCTGCCTCTATATCT	TT	TCCTTTATGCTCCGACACGAACATA	GTGCCTTAACGCAGTCGCTTGTCTC	TT	CCACTCAACTTTAACCCG
2	GTCCCTGCCTCTATATCT	TT	GAAATTTGACCACCGGTACCAGGCG	AGTCTTCCCAGTGCCTCTCGTCCTG	TT	CCACTCAACTTTAACCCG
3	GTCCCTGCCTCTATATCT	TT	CTCCCATTGACGAGTAGGTATCGCC	GAATGCCCAACTGAGATTGGACGTT	TT	CCACTCAACTTTAACCCG
4	GTCCCTGCCTCTATATCT	TT	CTGCGCTCCTGGTGGCGGTGGAGGT	GTTCATACTCATGTTGTACATGCCG	TT	CCACTCAACTTTAACCCG
5	GTCCCTGCCTCTATATCT	TT	TGGGTGGTTGGCACCATATTGTAGG	CTAATCATTTGACCCGGTTGCTGAC	TT	CCACTCAACTTTAACCCG
6	GTCCCTGCCTCTATATCT	TT	TCCTGAGCTTTGCCGATGTTCTTCT	TTTTTGGCTGCGTAGAGATTGGCTT	TT	CCACTCAACTTTAACCCG
7	GTCCCTGCCTCTATATCT	TT	TGGACACCTGCGAAACCGTGATACC	ACCTTATCCGTTTGTTCCCAAACCA	TT	CCACTCAACTTTAACCCG
8	GTCCCTGCCTCTATATCT	TT	TTCACTAGGATAGGGATTACTAAGA	TTTCCTCGCCAGTTCCTCTTTCGCC	TT	CCACTCAACTTTAACCCG
9	GTCCCTGCCTCTATATCT	TT	GTCGCCTGTTTACTGAAGTTTCTCC	GAATAGAAGTACTCGTTGAGGATCT	TT	CCACTCAACTTTAACCCG
10	GTCCCTGCCTCTATATCT	TT	TGAGGATCATGACGGCTTCGCAAGT	TCCGCCTGGCGTCCAAGAACCTGGA	TT	CCACTCAACTTTAACCCG
11	GTCCCTGCCTCTATATCT	TT	GAACTTCTTGTGAATAATTTGTACC	CTGCTTCAGCTGGACTTGTATAGAG	TT	CCACTCAACTTTAACCCG
12	GTCCCTGCCTCTATATCT	TT	CGCGTCCGACTTTGTTCCCGAAGCA	CGTTCAATCTCCTTCGGCGTAATCG	TT	CCACTCAACTTTAACCCG
13	GTCCCTGCCTCTATATCT	TT	ACGCCTGTTCATATTTTTCTAGTTC	TCATTACGTGCGTCGTGAACTCATT	TT	CCACTCAACTTTAACCCG
14	GTCCCTGCCTCTATATCT	TT	TTTTGCCCTGTAATCCGAATGTTCG	GTGATAAATTTGCCTAATTTGTGCA	TT	CCACTCAACTTTAACCCG

15	GTCCCTGCCTCT ATATCT	TT	GAAGCCGCTGCTGACGTA TTCGCCG	GCGTTTTCCGGCTGACCTG GTCCAC	TT	CCACTCAACTTTA ACCCG
16	GTCCCTGCCTCT ATATCT	TT	CGGCTACCCCTTCCGCTAT GAGCAT	CCCCTGCGCCTCCACCCTT TTCGGG	TT	CCACTCAACTTTA ACCCG
17	GTCCCTGCCTCT ATATCT	TT	AGGCTCTTCTTCTTGTGTG TTCCGT	ATCTAACCTCATTAGTTGT GGATCT	TT	CCACTCAACTTTA ACCCG
18	GTCCCTGCCTCT ATATCT	TT	GAACAGTGCTGGCTTCATA CGGTGA	TTTTTCCTTGATCTCGCAG AGCACA	TT	CCACTCAACTTTA ACCCG
19	GTCCCTGCCTCT ATATCT	TT	TCGTCCAAACTCTGGTCCG TAATAT	TTCAACGTATGCTTCCTGG CTTGCG	TT	CCACTCAACTTTA ACCCG

Table S13. Probe pairs designed for *Titanopuga salinarum dac* HCR in situ hybridization (B2 initiator).

Pair	Initiator	Spacer	Hybridization	Hybridization	Spacer	Initiator
1	CCTCGTAAATCCT CATCA	AA	TGTCCGTTAATTGTACTAC CGTGCA	TTTTTTCTCTGCCCATGTT GCGGGG	AA	ATCATCCAGTAA ACCGCC
2	CCTCGTAAATCCT CATCA	AA	CCAGGTGTTTGCGAAGTCC TTTTTT	GGGGAATGATTCGTGATT TGCGTGT	AA	ATCATCCAGTAA ACCGCC
3	CCTCGTAAATCCT CATCA	AA	TATACAGACATGGGGCAG TCTTGCA	ATGGTTGGATGGGAGGTT GACTTCA	AA	ATCATCCAGTAA ACCGCC
4	CCTCGTAAATCCT CATCA	AA	TCTCCATCTCCAATTCTTG CGTTAG	TCCTTTCAGCGTCCGGTCT GCTGCT	AA	ATCATCCAGTAA ACCGCC
5	CCTCGTAAATCCT CATCA	AA	GGAAGTGTTCCGAAGTGC CTCTTCG	CTCGTTGAGTAGACGTAG CGTTTCG	AA	ATCATCCAGTAA ACCGCC
6	CCTCGTAAATCCT CATCA	AA	AGCTGCTCGTGAAGCCGA CGCCGAC	TGCGTTCGCTTCTTCATCT CGGACT	AA	ATCATCCAGTAA ACCGCC
7	CCTCGTAAATCCT CATCA	AA	AGAGAATTCTCGTCCTCTG TTCTTC	TCTTCTCTTTCTTCAACCT CTTTTG	AA	ATCATCCAGTAA ACCGCC
8	CCTCGTAAATCCT CATCA	AA	TTCCCTTTCCCGTAAGATC TCCATC	TAACTGTTTCTCTAAGTTC TCCCTG	AA	ATCATCCAGTAA ACCGCC
9	CCTCGTAAATCCT CATCA	AA	CTTTCTTGCTGTCGGGCGT TGTCTG	AACTCCGCTTTCTCCAAGT TAATCT	AA	ATCATCCAGTAA ACCGCC
10	CCTCGTAAATCCT CATCA	AA	TAAGAAGTGTTTCTATGGA CGATAC	CTACCTTGAGCAAACCCT GGATGTT	AA	ATCATCCAGTAA ACCGCC
11	CCTCGTAAATCCT CATCA	AA	TCCTGTGCTCTGGAAAGGC GCCGAT	TGGATTCGTGGTTGCGCTC CCATCG	AA	ATCATCCAGTAA ACCGCC
12	CCTCGTAAATCCT CATCA	AA	GTACTACTGACGTCAGGGT TATCAC	CTGGAAGTGAGTCGATCG GAATTGG	AA	ATCATCCAGTAA ACCGCC

Table S14. Probe pairs designed for *Archegozetes longisetosus dac* HCR in situ hybridization (B2 initiator).

Pair	Initiator	Spacer	Hybridization	Hybridization	Spacer	Initiator
1	CCTCGTAAATCCT CATCA	AA	TCCGTTTGTTGTTGTTGCT GTTGCG	TAATTGCAAAACTTTTAAT CTACTG	AA	ATCATCCAGTAA ACCGCC
2	CCTCGTAAATCCT CATCA	AA	CTTGTTGGTTTTGAGTGAT TTGCTG	GGGGAGTCGGTGACGATT TCAGAGA	AA	ATCATCCAGTAA ACCGCC
3	CCTCGTAAATCCT CATCA	AA	CTGCTGCTGATGTATCTCG TGCTCC	ATTCTGTGTATTGTGTGAT ATCTGT	AA	ATCATCCAGTAA ACCGCC
4	CCTCGTAAATCCT CATCA	AA	CAAATGCTCTCTCATTTCT CGCTCC	TTTTTGTTCTTCATTCAGT TGTTTT	AA	ATCATCCAGTAA ACCGCC
5	CCTCGTAAATCCT CATCA	AA	AGTTCAGCTTTTTGCAAAT GCAATT	TCAAGTTGTGCCGCCAAC TCAGCCC	AA	ATCATCCAGTAA ACCGCC
6	CCTCGTAAATCCT CATCA	AA	CAATTGCCAAAAGCCCCT CAATATT	TTTGTTGTTGTCTAGCATT ATGAGC	AA	ATCATCCAGTAA ACCGCC
7	CCTCGTAAATCCT CATCA	AA	TTGTTGAGTTTGCTGTTGC TGTGGA	CAGCAATGTTTCCAAACA ATGTTCC	AA	ATCATCCAGTAA ACCGCC
8	CCTCGTAAATCCT CATCA	AA	TTGCGGTTTCACTCGCACC CCGTGT	GAGGAGTTGTCGAACTGT TTCGATT	AA	ATCATCCAGTAA ACCGCC
9	CCTCGTAAATCCT CATCA	AA	GCTGACAGGGTTGTCGCT ATTGTTA	ACACGATATACCGTTAAA TCCAGAA	AA	ATCATCCAGTAA ACCGCC
10	CCTCGTAAATCCT CATCA	AA	TCGTCGTCATCATCAGTAG TGTCAT	CTATTATTACTTTCCACGT CGTCTT	AA	ATCATCCAGTAA ACCGCC
11	CCTCGTAAATCCT CATCA	AA	TGCGCTGCTGGTGTTACTG CTGCTG	ATTATTATTATTGTTTGTA TTGTTG	AA	ATCATCCAGTAA ACCGCC
12	CCTCGTAAATCCT CATCA	AA	GTTACGAGAGCTTAAGTT AAGTGCG	GTTATTTCCACTACTATTG ACATTA	AA	ATCATCCAGTAA ACCGCC
13	CCTCGTAAATCCT CATCA	AA	TGTTGCTGTGAAAGTAAC CATAAAT	GAGTTCGGGACATGTTGT TGTTGCT	AA	ATCATCCAGTAA ACCGCC

14	CCTCGTAAATCCT CATCA	AA	CCAGAACCTTCCGATGTG GCGTTGT	GTTGGATTGAAATCTGAG GCCACGG	AA	ATCATCCAGTAA ACCGCC
15	CCTCGTAAATCCT CATCA	AA	CTGGAGGCGGACTTGCAT TTGAAAA	CATTGCTTCCGTGATGCGA AGCTAT	AA	ATCATCCAGTAA ACCGCC
16	CCTCGTAAATCCT CATCA	AA	TGGTGCGGTTCCAGCGCT GAAGTGT	CACAAGTTGTTGCTGTTGC TGAGGA	AA	ATCATCCAGTAA ACCGCC
17	CCTCGTAAATCCT CATCA	AA	TTCGCTGCTAGCATTGCTG CTGCGG	AAGAAAGGAAATGCGGCC GAATAAC	AA	ATCATCCAGTAA ACCGCC
18	CCTCGTAAATCCT CATCA	AA	TATTAAGACTGCTATGCGT TTGCTT	CGGCACTTGTGTTACCACT ACTATT	AA	ATCATCCAGTAA ACCGCC
19	CCTCGTAAATCCT CATCA	AA	ATCATCAGAGAAACGGGG CTTTTTA	GTCTATATTTTCACTGGCA TAGTCA	AA	ATCATCCAGTAA ACCGCC
20	CCTCGTAAATCCT CATCA	AA	TTGTGTTGCTGTGGCCCTG AGGTGA	GTTGGGTATCCGAGATAG GGCACAA	AA	ATCATCCAGTAA ACCGCC
21	CCTCGTAAATCCT CATCA	AA	TGACTGAACTTCTCTTGGG TGGACG	ATGATGGGACTGGGCTAA CAATGCC	AA	ATCATCCAGTAA ACCGCC

Table S15. Probe pairs designed for *Ixodes scapularis exd* HCR in situ hybridization (B2 initiator).

Pair	Initiator	Spacer	Hybridization	Hybridization	Spacer	Initiator
1	CCTCGTAAATCC TCATCA	AA	TTCCGAACTCTGCGGCGGA CTGTAG	TCACAGCGGACTGAGCGG GGTTGAG	AA	ATCATCCAGTAA ACCGCC
2	CCTCGTAAATCC TCATCA	AA	GATTGCACGTTGGCGCCCA TGGCGT	ATGGCATGCATGCCGTGG GGAAGCT	AA	ATCATCCAGTAA ACCGCC
3	CCTCGTAAATCC TCATCA	AA	TGTTGTACATGGAGTCCTG GGGCCC	GGTAGGAGTCGCCGCCGTT CATGCT	AA	ATCATCCAGTAA ACCGCC
4	CCTCGTAAATCC TCATCA	AA	GACCCTGCGTCGTGGGCAC CATGCT	GGGGCGGACTGATCATCT GACCCTG	AA	ATCATCCAGTAA ACCGCC
5	CCTCGTAAATCC TCATCA	AA	GCTGCGTACAGGTTTGCCT CTTCTT	TTTTTCTTTTTCGGGAAGA CTTTCT	AA	ATCATCCAGTAA ACCGCC
6	CCTCGTAAATCC TCATCA	AA	TCCGCTTGTTGCCGAACCA ATTAGA	CTTTCCCTATGTTCTTCTTG TATCG	AA	ATCATCCAGTAA ACCGCC
7	CCTCGTAAATCC TCATCA	AA	GGCGAGTTCCTCCTTGGCC TCCTCC	CTGAGAGACGGTGATGCC GCACTTC	AA	ATCATCCAGTAA ACCGCC
8	CCTCGTAAATCC TCATCA	AA	AAATACTCGTTCAGGATCT CCGTCG	GGGTACGGGTTGCTAAGG TGCGAGT	AA	ATCATCCAGTAA ACCGCC
9	CCTCGTAAATCC TCATCA	AA	TTGCGTCGAGGAACCTGGA CCTGAG	GCTTGCTGAAGTTGCGTCT CTTTCG	AA	ATCATCCAGTAA ACCGCC
10	CCTCGTAAATCC TCATCA	AA	GAGCTGCACCTGGATGGAG TTGAAC	CATGACGGCTTCGCAGGTG CTCTGC	AA	ATCATCCAGTAA ACCGCC
11	CCTCGTAAATCC TCATCA	AA	ATCTCCTTGGGCGTGATCG GCCGCG	TTGTGGATTATCTGCACCA TGCGCT	AA	ATCATCCAGTAA ACCGCC
12	CCTCGTAAATCC TCATCA	AA	CGTGCGTCGTGAACTCGTT GCACGC	GACTCTGCTCTCGCAAAAG GTTCAT	AA	ATCATCCAGTAA ACCGCC
13	CCTCGTAAATCC TCATCA	AA	GATCTGCCTGATCTGGGCG AGCTTG	CTCGTACTTCTCCAGCTCC TGGTGG	AA	ATCATCCAGTAA ACCGCC
14	CCTCGTAAATCC TCATCA	AA	CCGCTGCCCCGGCGCCGCC GCCCTT	CCCCGGAGGCCGCCGCAG ACGCGTT	AA	ATCATCCAGTAA ACCGCC

Table S16. Probe pairs designed for *Ixodes scapularis dac-A* HCR in situ hybridization (B3 initiator).

Pair	Initiator	Spacer	Hybridization	Hybridization	Spacer	Initiator
1	GTCCCTGCCTCTA TATCT	TT	ACCGTTTTGTTGCAACGAG ATCCTC	GCGGTCCGTACCTGTCGAT CGAAAG	TT	CCACTCAACTTTA ACCCG
2	GTCCCTGCCTCTA TATCT	TT	AGTGTATTGCACCCGGAAT CGAACG	AAGCGGATGGTTTTGCGG CTCGAGT	TT	CCACTCAACTTTA ACCCG
3	GTCCCTGCCTCTA TATCT	TT	GGCGTCTGGGTAATGCTGC GAAAGG	CGTCATCTATTGTGGTTTG AGGAAT	TT	CCACTCAACTTTA ACCCG
4	GTCCCTGCCTCTA TATCT	TT	CCCTGAACCAAATCGGTC GATGTCT	GCTGAAAGTGACGGCGCT GCGTTGC	TT	CCACTCAACTTTA ACCCG
5	GTCCCTGCCTCTA TATCT	TT	GCTTTCACCCCGTTCATAC CAATTA	TTTCCACGAACTGCTTTTG AAAGCA	TT	CCACTCAACTTTA ACCCG
6	GTCCCTGCCTCTA TATCT	TT	GAATCGTAAAGCGCTCAC TGTGTTT	TACGTAGGGAAGAACAGC CTTTGGC	TT	CCACTCAACTTTA ACCCG
7	GTCCCTGCCTCTA TATCT	TT	ATATTGCTTCTTGCAGTGG TGCAGT	AAAAACGAAACAGATGGA GGAACGG	TT	CCACTCAACTTTA ACCCG
8	GTCCCTGCCTCTA TATCT	TT	AGAGCAGCTTGCAGCGAT TCACCCC	TGTAGAGCGCGTCAAAGT CCTTGCA	TT	CCACTCAACTTTA ACCCG

9	GTCCCTGCCTCTA TATCT	TT	TTACACACGATCGGACTG ATGCCCA	CCCCGCAGCACGCGGACT TGCTCGA	TT	CCACTCAACTTTA ACCCG
10	GTCCCTGCCTCTA TATCT	TT	AACCGCCCACCAGGTGCC TGAGGAA	GCTTGAGCTTTGTGTACAC CGTGTG	TT	CCACTCAACTTTA ACCCG
11	GTCCCTGCCTCTA TATCT	TT	GTACTCGCCGCGGTACGC GAACGCC	CTCGAAGGCCTGGGGCAG GCAGAGC	TT	CCACTCAACTTTA ACCCG
12	GTCCCTGCCTCTA TATCT	TT	CTGCAGTCCGTCGCGTCGG CGGCCA	ATCTTCTCGCCGCGGTACT GGATGA	TT	CCACTCAACTTTA ACCCG
13	GTCCCTGCCTCTA TATCT	TT	CGTTGGACAAGGGGTCGA GACCCCG	ACCTCGACGAGGGACTCT TGAGCCT	TT	CCACTCAACTTTA ACCCG
14	GTCCCTGCCTCTA TATCT	TT	GCAGAGGGCGGCCGCGTT GAAAGCC	CCCGTTGATGACCGAGCA GAACTGG	TT	CCACTCAACTTTA ACCCG
15	GTCCCTGCCTCTA TATCT	TT	GACGGGGACGAGGAACCG CGGCTGT	GGGAGCGGTGGCGGCAGG TCGTGGG	TT	CCACTCAACTTTA ACCCG

Table S17. Probe pairs designed for *Ixodes scapularis dac-B* HCR in situ hybridization (B3 initiator).

Pair	Initiator	Spacer	Hybridization	Hybridization	Spacer	Initiator
1	GTCCCTGCCTCTA TATCT	TT	CGATCTGAATCAACTTCC GGTGACG	TTTTTGTCGGGTTCCTGTC TGCTTT	TT	CCACTCAACTTTA ACCCG
2	GTCCCTGCCTCTA TATCT	TT	TTGCGGTTGCTGCTGCTGC TGCGAT	GGACTCTGGACGTTGTAG AGGCTGC	TT	CCACTCAACTTTA ACCCG
3	GTCCCTGCCTCTA TATCT	TT	CCTCTTCGTACTGAGACCG TTTCTT	GCGGGAGTGTGGCCGTGG TTCGCAG	TT	CCACTCAACTTTA ACCCG
4	GTCCCTGCCTCTA TATCT	TT	CGCCGACAGCGGCGTTCT TTCTTGA	TCGAGTTCCAGCTGGTCCT GGATAC	TT	CCACTCAACTTTA ACCCG
5	GTCCCTGCCTCTA TATCT	TT	GCTCCTCCGTTAGCTGCTT TTCCAG	TCTTTTGGTAGAGCACCCT GGTCCG	TT	CCACTCAACTTTA ACCCG
6	GTCCCTGCCTCTA TATCT	TT	CTCCATCTTGAGCTCGGCC TTTTCG	TTCCCGAAGTCCTCGTTCT CGTAAG	TT	CCACTCAACTTTA ACCCG
7	GTCCCTGCCTCTA TATCT	TT	CACTTTGAGGAGTCCCTG GATGTTT	GTCTTGGTGCCTGGCGTTG TCGGCG	TT	CCACTCAACTTTA ACCCG
8	GTCCCTGCCTCTA TATCT	TT	ATTTCTTGGTCTCTGTCGT CGGTGT	GGGCTGCTGCTTTCTTCGC CGCCGC	TT	CCACTCAACTTTA ACCCG
9	GTCCCTGCCTCTA TATCT	TT	TCCGTGCGGTGAGCGTGT TGCGACA	GCGCCATCTTGGTATTCCC TAGGCT	TT	CCACTCAACTTTA ACCCG
10	GTCCCTGCCTCTA TATCT	TT	ATGCCGACGAAGACAGAC CACCATT	TCAGCACCGGAGGACCAT GTCCATT	TT	CCACTCAACTTTA ACCCG
11	GTCCCTGCCTCTA TATCT	TT	TCCATCGTAGGACGAGAA CATCCGG	GAAGGCAGCCGAGTCTTT GAGTCGG	TT	CCACTCAACTTTA ACCCG
12	GTCCCTGCCTCTA TATCT	TT	CCGTCTCGCTCTTGTTGTG ATCAAA	CGCTGCCGTGGGGCTTGTC CGCATG	TT	CCACTCAACTTTA ACCCG
13	GTCCCTGCCTCTA TATCT	TT	TGCACCACCATCAACGTG GTCGAGT	CACCTCAGGTACCGATACC GACACG	TT	CCACTCAACTTTA ACCCG
14	GTCCCTGCCTCTA TATCT	TT	CCTTTTCGATGCTGATCTG TCGGTC	GTGGCGATGATTCCAAAG TCCACTG	TT	CCACTCAACTTTA ACCCG
15	GTCCCTGCCTCTA TATCT	TT	AGGAGTCCCTGGATGTTT CGAAGCA	TGCCTGGCGTTGTCGGCGG CCACTT	TT	CCACTCAACTTTA ACCCG
16	GTCCCTGCCTCTA TATCT	TT	CACCGCCGCTCATTTCTTG GTCTCT	CATGGGGTCCCGGGCTGCT GCTTTC	TT	CCACTCAACTTTA ACCCG
17	GTCCCTGCCTCTA TATCT	TT	GTGCGGTGAGCGTGTTGC GACAGGT	CCATCTTGGTATTCCCTAG GCTCGT	TT	CCACTCAACTTTA ACCCG
18	GTCCCTGCCTCTA TATCT	TT	CCGACGAAGACAGACCAC CATTGAT	GGACCGGAGGACCATGTC CATTGGA	TT	CCACTCAACTTTA ACCCG
19	GTCCCTGCCTCTA TATCT	TT	ATCGTAGGACGAGAACAT CCGGTCC	GGCAGCCGAGTCTTTGAGT CGGCTT	TT	CCACTCAACTTTA ACCCG
20	GTCCCTGCCTCTA TATCT	TT	TCGATGCGCCCCTTCTTGC TCATCA	TGACCGTTCTCGTAGCCGG GGTAGT	TT	CCACTCAACTTTA ACCCG
21	GTCCCTGCCTCTA TATCT	TT	TGGAATGTGTTTCCCGTTT GGGGAA	GGGGACGGGCTTGGTTCG CAACTCG	TT	CCACTCAACTTTA ACCCG
22	GTCCCTGCCTCTA TATCT	TT	TCGATGCGCCCCTTCTTGC TCATCA	TGACCGTTCTCGTAGCCGG GGTAGT	TT	CCACTCAACTTTA ACCCG
23	GTCCCTGCCTCTA TATCT	TT	GGTAGACCCTGGTAGCTT CTCCGAA	GACGGGCTTGGCGGGTCT GGAAACT	TT	CCACTCAACTTTA ACCCG
24	GTCCCTGCCTCTA TATCT	TT	TGCAGTCCTTGTAGAGCG CATCAAA	ACAGGCGGCTGGCAGATC TCGCCGT	TT	CCACTCAACTTTA ACCCG
25	GTCCCTGCCTCTA TATCT	TT	ACGATCGGCGTGATGTCC AGCCTCT	AGGATCCTGACCTGCTCGA CGTTGC	TT	CCACTCAACTTTA ACCCG
26	GTCCCTGCCTCTA TATCT	TT	ACCAGGTGCTTGAGGAAC AGCTCGA	TTCGTGTACACCGTGTGCA GCCCTC	TT	CCACTCAACTTTA ACCCG

Table S18. Probe pairs designed for *Pycnogonum litorale exd* HCR in situ hybridization (B1 initiator).

Pair	Initiator	Spacer	Hybridization	Hybridization	Spacer	Initiator
1	GAGGAGGGCAGCAAACGG	AA	GCCTAAAGCTGCCTAATTTAACAAA	AAAAATAAGCTCGCATCGTTTTTAA	TA	GAAGAGTCTTCCTTTACG
2	GAGGAGGGCAGCAAACGG	AA	TTAAAACAATGTTGCCATTAATTCA	TTTTTAAAAGTGGCGAGGTTTCATC	TA	GAAGAGTCTTCCTTTACG
3	GAGGAGGGCAGCAAACGG	AA	ACAGTCAGCCAAATAATAAATATTT	TTTGTAAAATTATATTAAATATTAT	TA	GAAGAGTCTTCCTTTACG
4	GAGGAGGGCAGCAAACGG	AA	ATTATTTATTTAAAATTTTAATAAC	AAAAATCTTTACGTTTATGATACCT	TA	GAAGAGTCTTCCTTTACG
5	GAGGAGGGCAGCAAACGG	AA	ATCTTTTAAATTGTATTTTAACACA	AAAAATTTTTCAAAACAGAATCCAA	TA	GAAGAGTCTTCCTTTACG
6	GAGGAGGGCAGCAAACGG	AA	TAAATATATATACTTGGTTTGTTAC	AAAAATAAAGGTATCATAACGTCAT	TA	GAAGAGTCTTCCTTTACG
7	GAGGAGGGCAGCAAACGG	AA	TCACTAGGACTGGCTGCAGATTGTG	TGTGGTGGGACGCTATTGGCTGACA	TA	GAAGAGTCTTCCTTTACG
8	GAGGAGGGCAGCAAACGG	AA	GGTGCATATCGTACATCCCACCTGA	TCGACGAATGATTATATCCTCCAAC	TA	GAAGAGTCTTCCTTTACG
9	GAGGAGGGCAGCAAACGG	AA	TCCAGTCTGTGATTGTACGTTGGCA	ATGTGCTGGTAATCCGTCTGAGTAT	TA	GAAGAGTCTTCCTTTACG
10	GAGGAGGGCAGCAAACGG	AA	CCTCCATTCATACTCATACTTCCAG	ATTGAAGAAATTTGTTGGTAAGAAT	TA	GAAGAGTCTTCCTTTACG
11	GAGGAGGGCAGCAAACGG	AA	TCATTTGACCTTGACCCTGTGATGA	ACATCTGGTCACCCGGAGGAGGACT	TA	GAAGAGTCTTCCTTTACG
12	GAGGAGGGCAGCAAACGG	AA	GACCTCAGTATCATGACGGCTTCGC	TTTTTCCTCCTAGCATCTAAGAACC	TA	GAAGAGTCTTCCTTTACG
13	GAGGAGGGCAGCAAACGG	AA	TCTATTTCTTTCGGCGTAATTGGCC	TTTTTGTGAATAATTTGGACCATTC	TA	GAAGAGTCTTCCTTTACG
14	GAGGAGGGCAGCAAACGG	AA	TCACGTGCGTCGTAAACTCGTTACA	TTCTACTTTGTTCTCTCAGAAGGTT	TA	GAAGAGTCTTCCTTTACG
15	GAGGAGGGCAGCAAACGG	AA	GTATATTTGTCGAATTTGAGCTAAT	CTGTTCGTATTTTTCCAATTCCTGA	TA	GAAGAGTCTTCCTTTACG
16	GAGGAGGGCAGCAAACGG	AA	CTGTCCGGTTGTCCAGGACCACCTG	GCGCGATAATCGGAATGCTCGATAG	TA	GAAGAGTCTTCCTTTACG
17	GAGGAGGGCAGCAAACGG	AA	CAGCACCTCCGCCTTTTTCTGGACC	CTGCAGCCGATGCATTAGCCGCCGC	TA	GAAGAGTCTTCCTTTACG
18	GAGGAGGGCAGCAAACGG	AA	CAACCGCATTAGTTGTGGATCGGGC	GACACCTTCCGCTATTAACATGTTA	TA	GAAGAGTCTTCCTTTACG
19	GAGGAGGGCAGCAAACGG	AA	GAACAATGCCGGTTTCATACGATGA	TTTTTCTTTAATTTCGCACAATACA	TA	GAAGAGTCTTCCTTTACG
20	GAGGAGGGCAGCAAACGG	AA	TCATCTAAGCTTTGATCGGTAATGT	TTGAGTGTATGTTTTCTCGCTTGCG	TA	GAAGAGTCTTCCTTTACG
21	GAGGAGGGCAGCAAACGG	AA	CTTGTTTACGGCCATCCTGATCGTG	TAATTTGTTGTAATATTTCACCAAT	TA	GAAGAGTCTTCCTTTACG
22	GAGGAGGGCAGCAAACGG	AA	TGATGGGGCTTGAAGACCGTATCCA	CGGTGGTTGATGGCCTGGATCACCG	TA	GAAGAGTCTTCCTTTACG
23	GAGGAGGGCAGCAAACGG	AA	ACAACCGCATGGCCGTGCTGCTGCA	TGAGGTACGACATGACCAGCCATAC	TA	GAAGAGTCTTCCTTTACG
24	GAGGAGGGCAGCAAACGG	AA	TTACTCGACTAATGTATAAATCTAG	TTCTTTGTTGCTCGTCCATATCCTA	TA	GAAGAGTCTTCCTTTACG
25	GAGGAGGGCAGCAAACGG	AA	AAAACTATTCAATACACTAACAGCG	AAAAACTAGCGAAGGAGAGAACGAC	TA	GAAGAGTCTTCCTTTACG
26	GAGGAGGGCAGCAAACGG	AA	CTTCAATCCACGACACACTCACACT	ACTTTATCGTAGTAGTCACTGCTTC	TA	GAAGAGTCTTCCTTTACG

Table S19. Probe pairs designed for *Pycnogonum litorale dac* HCR in situ hybridization (B5 initiator).

Pair	Initiator	Spacer	Hybridization	Hybridization	Spacer	Initiator
1	CTCACTCCCAATCTCTAT	AA	CCGTAATATTTACAATTTATATTTA	GTTCAACAATAATACTACTTTATAA	AA	CTACCCTACAAATCCAAT
2	CTCACTCCCAATCTCTAT	AA	TGAAATTATGAAACGACCCCTACGT	ATGAAGAATCTTTTTCGCGTTTTAA	AA	CTACCCTACAAATCCAAT
3	CTCACTCCCAATCTCTAT	AA	GCCAACGATTCGTTAAGTATTCTTA	TTGTTTCTGTCCCGTTCTATGTCGT	AA	CTACCCTACAAATCCAAT
4	CTCACTCCCAATCTCTAT	AA	CTGATCCTTTGTTCTTCCAACAATT	TTTTTGATCCGTTTTTGGTACAAAA	AA	CTACCCTACAAATCCAAT
5	CTCACTCCCAATCTCTAT	AA	TTGCCTGGCATTGTCGGCTGCCACT	TTTCTCGTAGTTGCTTTGTCGTTCT	AA	CTACCCTACAAATCCAAT

6	CTCACTCCCAATCTCTAT	AA	TGTTGTTCAACATTCCGACAGCTTG	CGGCCGTCATGGTGCCCATGCCAGG	AA	CTACCCTACAAATCCAAT
7	CTCACTCCCAATCTCTAT	AA	TCTTCTTCGGTGTCGTTTTCGTCTT	AAATCGTGTTCTCTGTCGTCGTCGT	AA	CTACCCTACAAATCCAAT
8	CTCACTCCCAATCTCTAT	AA	CTTCGCTACCACTGTGTTCTGTTCC	CGTCTTCTCCGACGTCATTGAAAGC	AA	CTACCCTACAAATCCAAT
9	CTCACTCCCAATCTCTAT	AA	TTGTGACAGATTTAATACTGAGCTG	ATTTCCGCCGCCATGGTTACGGTTG	AA	CTACCCTACAAATCCAAT
10	CTCACTCCCAATCTCTAT	AA	GAATCTTTGATCCTGTGGTGCTCGT	CCATTCGTCGAACCGTTCGCGAATG	AA	CTACCCTACAAATCCAAT
11	CTCACTCCCAATCTCTAT	AA	TGTCGCTCGATTCGCATCGTTCTTC	ATGCAGGCGAACTGTAAAGTCGCTC	AA	CTACCCTACAAATCCAAT
12	CTCACTCCCAATCTCTAT	AA	GGCGCTACTGATGGCCGAACGGTGG	TGATCTGGATGAATTGATGGCGTCG	AA	CTACCCTACAAATCCAAT
13	CTCACTCCCAATCTCTAT	AA	CTGGTCGTTGGTAGGAGTCCTGACA	TTGTTGGAAGTGACGTCATTGGATC	AA	CTACCCTACAAATCCAAT
14	CTCACTCCCAATCTCTAT	AA	GACTAGAATTTGATAATGCGCTCGG	GGTGGTGGTGATGATGATGATTTGG	AA	CTACCCTACAAATCCAAT
15	CTCACTCCCAATCTCTAT	AA	TCCACCTTGATGATGGTGGTGGTGA	TAACAGACTCGATGCTCCACCACCA	AA	CTACCCTACAAATCCAAT
16	CTCACTCCCAATCTCTAT	AA	ATCGGTATGAATGGTAAAGAATTGA	CCTCCGTGGACACCATGATGACTTG	AA	CTACCCTACAAATCCAAT
17	CTCACTCCCAATCTCTAT	AA	CTGCATGGTGACTGTAACCATTGGC	GTGCAGCTGCAGCTGCTACCTGGGC	AA	CTACCCTACAAATCCAAT
18	CTCACTCCCAATCTCTAT	AA	TGCACGAAGAAATTCGGCTGTGATG	CAATGGATTCTTCTCCAATTGTTCG	AA	CTACCCTACAAATCCAAT
19	CTCACTCCCAATCTCTAT	AA	TTATCAATGCGACATTTCTTGGCCT	CCGTTTTCGTATCCCAGGTAATCTC	AA	CTACCCTACAAATCCAAT
20	CTCACTCCCAATCTCTAT	AA	GGCTCATACCGTCAATTAATGAGGC	TTATTGCGCTATCAGATGGATGTGT	AA	CTACCCTACAAATCCAAT
21	CTCACTCCCAATCTCTAT	AA	CCGATTGAGCCAACGCCACCGCCAC	TTTTTAGCACTAGGGTCACAATGAC	AA	CTACCCTACAAATCCAAT
22	CTCACTCCCAATCTCTAT	AA	AAACCGATGGCCGATGATGGTGATG	CACCTGTAGATTGCGGTGGACTGTT	AA	CTACCCTACAAATCCAAT
23	CTCACTCCCAATCTCTAT	AA	CATACTCGGAACCATGCTCGATATA	ATGGTGATGCTGTTCTAATGCTTTG	AA	CTACCCTACAAATCCAAT
24	CTCACTCCCAATCTCTAT	AA	TTCGAAGCCACAGACGCTGCTAAAG	GAAGCACTGAGTGCTCCGTACGGGA	AA	CTACCCTACAAATCCAAT
25	CTCACTCCCAATCTCTAT	AA	CGTTATCGCCGTGAGACGAAGTGAC	GCGGTAACATCGGCGGCGATGGACT	AA	CTACCCTACAAATCCAAT
26	CTCACTCCCAATCTCTAT	AA	TGACATTGGACTCGTACTATTCTCG	AGTCGTGAGGCCAGTGGCAGGACCA	AA	CTACCCTACAAATCCAAT
27	CTCACTCCCAATCTCTAT	AA	TCCAGTGCTCCTATCGATGGAGGCT	GGCGACGGTTGCTTCATGTTCAGCA	AA	CTACCCTACAAATCCAAT
28	CTCACTCCCAATCTCTAT	AA	TCAGCTGACCATTGGTACTCGGAAG	GCGGCGATCCGAGAGGCGTCGACGG	AA	CTACCCTACAAATCCAAT
29	CTCACTCCCAATCTCTAT	AA	CGACGACGATGCAGCATCAGTTTGA	CGGCGGCGGCGACGGCGACGGCGAA	AA	CTACCCTACAAATCCAAT
30	CTCACTCCCAATCTCTAT	AA	ATGATGAATGAATGAATGAATAATG	CCAGAGCTAACAGGCTGCTGCTGCT	AA	CTACCCTACAAATCCAAT

Table S20. Primer pairs used in RNAi experiments. Primer pairs for *Po-dac* were previously published in Sharma et al. (2013).

Primer ID	Forward Primer	Reverse Primer
Po-exd	5'-GCGCAAGCTAGGAAACACAC-3'	5'-ATTCCTCCTTGGCTTCTTCG-3'
Po-N	5'-CGGTTTCAAAGGTGTCGATT-3'	5'-TGACGCAAGGATTGCTGTAG-3'

Supplementary References

Börner C. 1901. Zur äusseren Morphologie von *Koenenia mirabilis* Grassi. *Zoologischer Anzeiger* 24:537-556.

Chamberlin JC. 1931. The arachnid order Chelonethida. *Stanford University Publications, Biological Sciences* 71:1-284.

Cook DR. 1974. Water mite genera and subgenera. *Memoirs of the American Entomological Institute* 21:1-860.

Evans GO. 1992. Principles of Acarology. CAB International, Wallingford, Oxfordshire, UK.

Foelix RF. 2011. Biology of Spiders. Oxford University Press, New York, New York.

Graveley FH. 1915. Notes on the habits of Indian insects, myriapods, and arachnids. *Rec. Indian Mus.* 9:483-539.

Harvey MS. 1992. The phylogeny and classification of the Pseudoscorpionida (Chelicerata: Arachnida). *Invertebrate Taxonomy* 6:1373-1435.

Helfer H, Schlottke E. 1935. Pantopoda. Dr. H.G. Bronns Klassen und Ordnung des Tierreichs, Band 5, Abt. IV, Buch2. Leipzig: Akademische Verlagsgesellschaft m.b.H.

Hjelle JT. 1990. Anatomy and morphology. In: G.A. Polis, editor. The Biology of Scorpions: 9-63. Stanford University Press, Stanford, California.

King PE. 1973. Pycnogonids. Hutchinson, London, UK.

Kraepelin K. 1891. Scorpiones und Pedipalpi. *Tierrich.*

Krantz GW, Walter DE, Behan-Pelletier V, Cook DR, Harvey MS, Keirans JE, Lindquist EE, Norton RA, OConnor BM, Smith IM. 2009. A Manual of Acarology. 3rd ed. (Krantz GW, Walter DE, editors). Texas Tech University Press, Lubbock, Texas.

Meinert F. 1899. Pycnogonida. In: The Danish Ingolf-Expedition. 3rd ed. p. 1–71.

Millot J. 1949a. Ordre des amblypyges. In: P.-P. Grassé, ed. *Traité de zoologie* 6:563-588. Masson, Paris, France.

Millot J. 1949b. Ordre des palpigrades. In: P.-P. Grass Grassé, ed. *Traité de zoologie* 6:520-532. Masson, Paris, France.

Millot J. 1949c. Ordre des ricinuléides. In: P.-P. Grassé, ed., *Traité de zoologie* 6:744-760. Masson, Paris, France.

Millot J. 1949d. Ordre des uropyges. In: P.-P. Grassé, ed., *Traité de zoologie* 6:533-562. Masson, Paris, France.

Millot J, Vachon M. 1949a. Ordre des scorpions. In: P.-P. Grassé, ed*., Traité de zoologie* 6:386-436. Masson, Paris, France.

Millot J, Vachon M. 1949b. Ordre des solifuges. In: P.-P. Grassé, ed*., Traité de zoologie*

6:482-519. Masson, Paris, France.

Pittard K, Mitchell RW. 1972. Comparative morphology of the life stages of *Cryptocellus pelaezi* (Arachnida, Ricinulei). *Graduate Studies, Texas Tech University* 1:1-78.

Punzo F. 1998. The Biology of Camel-Spiders (Arachnida, Solifugae). Norwell, Massachusetts 02061 USA: Kluwer Academic Publishers.

Rowland JM. 1975. Classification, phylogeny and zoogeography of the American arachnids of the order Schizomida. Texas Tech University.

Sars GO. 1891. Pycnogonidea. In: Norweigan North-Atlantic Expedition, 1876-1878. Christiana, Grøndahl & Søns bogtrykkeri. p. 1-163.

Savory T. 1964. Arachnida. Academic Press Inc., London, UK.

Schram FR, Hedgpeth JW. 1978. Locomotory mechanisms in Antarctic pycnogonids. *Zoological Journal of the Linnean Society* 63:145-169.

Sharma PP, Schwager EE, Extavour C, Giribet G. 2013. Evolution of the chelicera: A *dachshund* domain is retained in the deutocerebral appendage of Opiliones (Arthropoda, Chelicerata). *Evol & Development* 14(6):522-533.

Shultz JW. 1989. Morphology of locomotor appendages in Arachnida: evolutionary trends and phylogenetic implications. *Zoological Journal of the Linnean Society* 97:1-56.

Shultz JW. 1993. Muscular anatomy of the giant whipscorpion *Mastigoproctus giganteus* (Lucas) (Arachnida: Uropygi) and its evolutionary significance. *Zoological Journal of the Linnean Society* 108:335-365.

Shultz JW, Pinto-da-Rocha R. 2007. Morphology and Functional Anatomy. In: Pinto-da-Rocha R, editor. *The Biology of Opiliones.* Harvard University Press, Cambridge, Massachusetts.

Snodgrass RE. 1958. Evolution of arthropod mechanisms. Smithsonian Miscellaneous Collections 138(2), The Smithsonian Institution, Washington DC, USA.

van der Hammen L. 1966. Studies on Opilioacarida (Arachnida). I. Description of *Opilioacarus texanus* (Chamberlin and Mulaik) and revised classification of the genera. *Zoologische Verhandelingen* 86:1-80.

van der Hammen L. 1982. Comparative studies in Chelicerata II. Epimerata (Palpigradi and Actinotrichida). *Zoologische Verhandelingen* 196:1-70.

Weygoldt P. 1969. The biology of pseudoscorpions. Harvard University Press, Cambridge, Massachusetts.

Weygoldt P. 2000. Whip spiders (Chelicerata: Amblypygi): Their biology, morphology and systematics. Apollo Books, Strenstrup, Denmark.
